# Genus *Salsola*: Chemistry, Biological Activities and Future Prospective—A Review

**DOI:** 10.3390/plants11060714

**Published:** 2022-03-08

**Authors:** Samar S. A. Murshid, Dana Atoum, Dina R. Abou-Hussein, Hossam M. Abdallah, Rawan H. Hareeri, Haifa Almukadi, RuAngelie Edrada-Ebel

**Affiliations:** 1 Department of Natural Products and Alternative Medicine, Faculty of Pharmacy, King Abdulaziz University, Jeddah 21589, Saudi Arabia; samurshid@kau.edu.sa; 2 Strathclyde Institute of Pharmacy and Biomedical Sciences, University of Strathclyde, Glasgow G4 0RE, UK; dana.atoum@strath.ac.uk (D.A.); ruangelie.edrada-ebel@strath.ac.uk (R.E.-E.); 3 Department of Pharmacoagnosy, Faculty of Pharmacy, Cairo University, Cairo 11562, Egypt; dina.abouhussein@pharma.cu.edu.eg; 4 Department of Pharmacology and Toxicology, Faculty of Pharmacy, King Abdulaziz University, Jeddah 21589, Saudi Arabia; rhhareeri@kau.edu.sa (R.H.H.); hsalmukadi@kau.edu.sa (H.A.)

**Keywords:** genus *Salsola*, phytoconstituents, biological activities, Russian thistle, halophyte

## Abstract

The genus *Salsola* L. (Russian thistle, Saltwort) includes halophyte plants and is considered one of the largest genera in the family Amaranthaceae. The genus involves annual semi-dwarf to dwarf shrubs and woody tree. The genus *Salsola* is frequently overlooked, and few people are aware of its significance. The majority of studies focus on pollen morphology and species identification. *Salsola* has had little research on its phytochemical makeup or biological effects. Therefore, we present this review to cover all aspects of genus *Salsola,* including taxonomy, distribution, differences in the chemical constituents and representative examples of isolated compounds produced by various species of genus *Salsola* and in relation to their several reported biological activities for use in folk medicine worldwide.

## 1. Introduction

**Figure 1:**
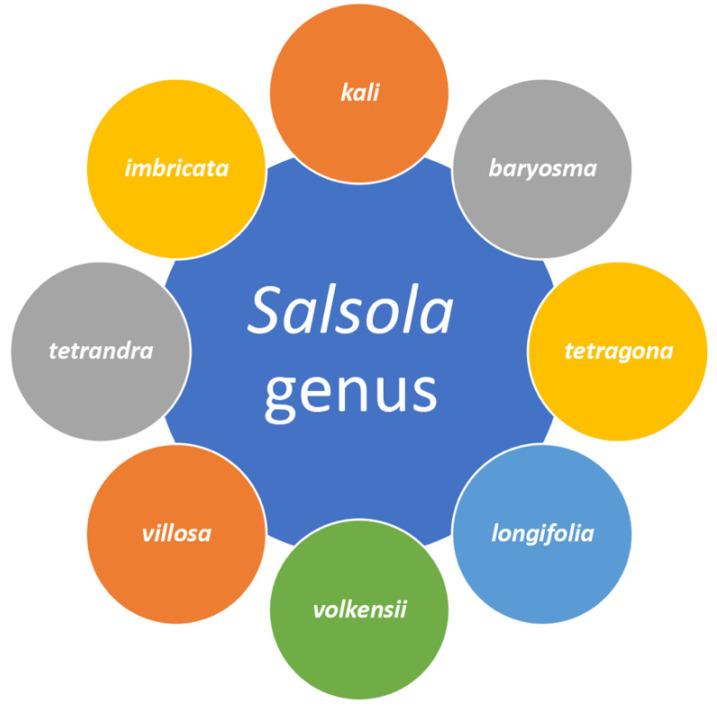
Representative example of the most-studied species of the genus *Salsola.*

The genus *Salsola* L. (Russian thistle, Saltwort), a genus of from semi-dwarf to dwarf shrubs and woody tree species, is a halophyte plant, which is considered one of the largest genera in the family Amaranthaceae. The genus can also help with the restoration and reclamation of degraded salty areas and saline soils [1,2,3,4]. The genus name derives from the Latin word *salsus*, which means “salty”, in reference to the salt-tolerant plants [5,6]. Moreover, this genus is recognized as a cosmopolitan group of plants, which are distributed and naturalized worldwide. The exact number of species that belong to this genus has yet to be determined. Over 64 species have been reported, which are widespread in arid and semi-arid regions of Central Asia, Middle East, Africa, and Europe (Figure 1) [3,7,8,9]. *Salsola* species have a variety of features that contribute to their recognition as a potential forage species in from semi-arid to dry settings along sea beaches, such as extensive seed production, and resistance to extreme climatic conditions including high temperature and extended drought conditions [8,10,11]. These plants typically grow on flat, generally dry and/or slightly saline soils, with some species occurring in salt marshes. Easy-to-vegetates on dry soil and is resistant to pH fluctuation and harsh weather. *Salsola* is found to be an allelopathically active species, which also decreased the growth of selected associated species during its decaying process [12]. It is autotoxic, but its germination is not inhibited by any of the isolated phytotoxins applied [13].

The genus is rich in vast classes of phyto-constituents, mainly flavonoids, phenolic compounds, nitrogenous compounds, saponins, triterpenes, sterols, volatile constituents, lignans, coumarins and cardiac glycosides. Moreover, it shows different biological activities, including analgesic, anti-inflammatory, antiviral, antibacterial, anticancer, cardioprotective and hepatoprotective activities. The genus *Salsola* is frequently overlooked, and few people are aware of its significance. The majority of studies focus on pollen morphology [14] and species identification [15] while little research has looked at its phytochemical makeup or biological effects. There is very little information on the adaptation characteristics of *Salsola* plants for their efficient use in drought-prone, semi-arid to arid settings, as well as their uses re-mediating degraded salt soils.

Therefore, we present this review to cover all aspects of the genus *Salsola* including taxonomy, distribution, chemical constituents and reported biological activities. This review is based on the literature obtained through a computer search in different databases, including ScinceDirect, Web of Knowledge, SCOPUS, Pub Med and Google Scholar, using the keywords “*Salsola* and chemistry”, “*Salsola* and phyto-constituents”, “*Salsola* and taxonomy” and “*Salsola* and biological activities”, from 2010 to 2021.

## 2. Taxonomy and Distribution

From the taxonomic perspective, *Salsola* belongs to tribe Salsoleae of subfamily Salsoloideae in family Amaranthaceae [16]. It includes about 64 species (Table 1) but, due to the physical similarity between many species, this genus is generally regarded as exceedingly tough [17,18]. Many writers researched the anatomy of the genus *Salsola*; however, they all focused on C3–C4 Kranz anatomy in the genus and allied genera’s leaves [19]. Mostly, *Salsola* species are shrubs, subshrubs, or trees. The leaves are alternate, small, simple, entire, and sessile. They are usually succulent, hairy, and thickly packed, which helps to protect the branches [20]. The genus’ stem anatomy was unusual and has been studied in few species, such as *S. kali* and *S. crassa* (synonym of *Climacoptera crass*) [18,19,21]. This is because of the difficulties in sectioning the woody, hard stem, as well as the aberrant secondary growth seen in many Amaranthaceae species [22].

Furthermore, *Salsola* leaves are classified into two anatomical types: the Salsoloid-type leaf, with continuous layers of chlorenchymatous cells with a vascular bundle at the center of the leaf and small peripheral vascular bundles that adhere to chlorenchyma [22], and Sympegmoid-type leaves, with two or three palisade layers and a discontinuous layer of indistinctive bundle sheath cells (typically non-Kranz) around water-storage tissue [22]. Flowers are bisexual, with five petals, five stamens, and a pistil with two stigmas. Finally, fruit is spherical, carrying seeds with a spiral embryo [9].

**Figure 2:**
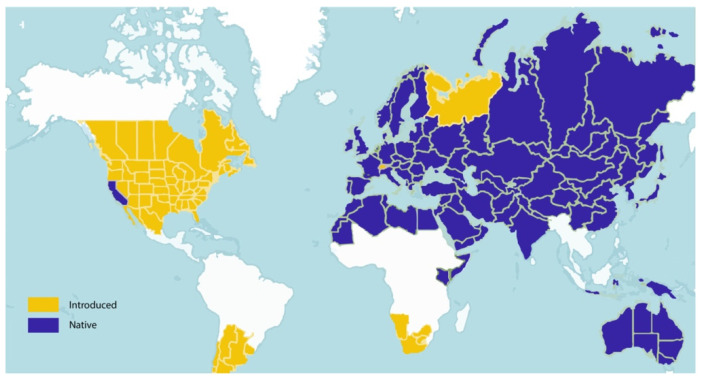
Distribution of genus *Salsola* in different regions of the world.

The genus resists soil salinity; therefore, it is known to grow in hypersaline, arid and semiarid regions [23]. The genus is native to Africa (Mediterranean region), Euro Asia, California, and Australia (Figure 2) [18]. It was introduced to South Africa, and some territories in North and South America [4].

## 3. Traditional Uses of Genus *Salsola*

Plants from the genus *Salsola* are known to be used in traditional medicine in treatment of different aliments. *S. somalensis* is used as hypotensive, antibacterial, anticancer agents and frequently used in traditional medicine to treat a variety of conditions, such as skin diseases and cure tape worm infestation [24,25,26]. In addition, the dried roots of *S. somalensis* are sold as an anthelmintic by conventional medication distributors in a variety of markets in Ethiopia [27]. The buds of *S. soda*, the main edible parts of the plant, are consumed as vegetables in Italy and called “agretti” or “barba di frate”. The plant was once utilized as a source of impure sodium carbonate, which gave it the name “soda” [26]. Other species, such as *S. tragus* and *S. baryosoma,* are utilized as livestock fodder in arid and dry areas [4]. The whole plant of *S. kali* is used as an infusion by indigenous people residing in the Rif region, Northern Morocco to treat digestive system disorders [28]. The local population in the Mongolian People’s Republic traditionally used the *S. laricifolia* herb for the treatment of stomach diseases, fractured bones, healing wounds, itching, and swelling joints [29,30]. In sheep, some members of the *Salsola* genus produce prolonged gestation (pregnancy), and, in female rats, they cause contraception (birth control) [31]. Bushmen women in Namibia and South Africa consume the aqueous extracts (tea infusion) of *S. tuberculatiformis* (synonym of *Caroxylon tuberculatiforme* (Botsch.) Mucina) as an oral contraceptive in traditional medicine by inhibiting the P450c11 and reducing the biosynthesis of corticosteroids [32]. Meanwhile, in the Cholistan desert, Southern Punjab, Pakistan, *S. baryosma* has a folklore reputation for treating indigestion, diarrhea, dysentery, itching, sores, colds, improve maleness, asthma, migraine, headache, and inflammations [14,17,20]. Moreover, in the Middle East, *S. baryosma* is used against some inflammatory diseases [33]. *S. imbricata* has several folk medicinal applications in the treatment of painful and inflammatory conditions [22], where the bark extract showed a higher potency than fruit extract as an anthelmintic [34]. In China and Korea [34,35,36], the whole fresh herb *of S. collina* is widely used to treat hypertension [35], headache, insomnia, constipation [37,38] and as a herbal drink or medicine [34,35]. In Russia, *S. collina* was a component of the biologically active food additive “Heparon”, which is recommended as a hepatoprotective when the hepatic cells are exposed to alcohol, medications and various toxins [38]. Hyperpyrexia, hypertension, inflammation, jaundice, and gastrointestinal illnesses have all been treated using *S. komarovii* in the past [37]. In addition, Bedouins and locals alike are familiar with *S. cyclophylla*, an edible halophyte, and its traditional medical usage in the treatment of inflammation and pain [36], as well as its other health benefits, including nutritional values [36,39]. Thus, the plant is used as a tea and concoction for medicinal purposes by both tribes and traditional healers to treat many diseases, particularly inflammation and pain. The plant is also used as a diuretic, laxative, and anthelmintic by the locals [40].

To find novel medications from the identified genus, more phytochemical, pharmacological, and toxicological research should be carried out.

## 4. Chemistry of *Salsola*

**Figure 3:**
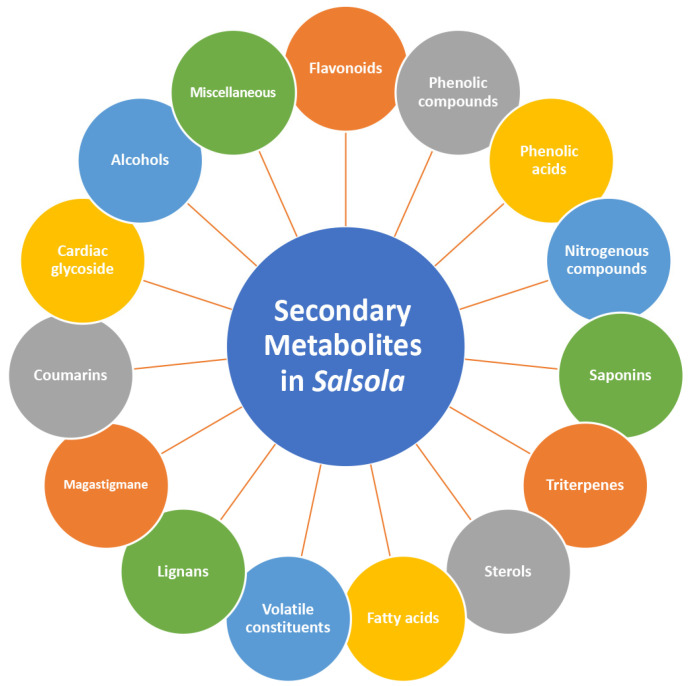
Different chemical constituents in genus *Salsola*.

Phytochemical composition and biological consequences of the genus have received very little attention. Only a few species from the genus *Salsola* have been chemically and biologically examined. The secondary metabolites in *Salsola* include flavonoids (**1**–**53**), phenolic compounds (**54**–**70**), phenolic acids (**71**–**95**), nitrogenous compounds (**96**–**126**), saponins (**127**–**137**), triterpenes (**138**–**144**), sterols (**145**–**151**), fatty acids (**152**–**186**), volatile constituents (**187**–**195**), lignans (**196**–**200**), magastigmane (**201**–**207**), coumarins (**208**–**219**), cardiac glycosides (**220**–**224**), alcohols (**225**–**228**) cyanogenic, isoprenoid, and sulphur containing compounds (**229**–**231**) (Figure 3). Flavonoids, phenolic compounds, and phenolic acids predominates in most of species in this genus. Volatile constituents were only examined in *S. vermiculata* and *S. cyclophylla*. Meanwhile, lignans and magastigmanes were only isolated from *S. komarovii*. On the other hand, cardiac glycosides were only isolated from *S. tetragona*. The authors found that the naming of many active compounds in *Salsola* was very confusing. Some active compounds were given a name derived from the genus, such as salcolin A (**23**) and B (**24**) (flavonoid nucleus), Salsoline A (**114**) and B (**115**) (nitrogenous compound) and Salsolin A (**142**) and B (**143**) (triterpene nucleus). Some isolated compounds were given confusing common names, for example, Biphenol 2 (**54**) was given to hydroxy tyrosol-4′—glucopyranoside [41]. Moreover, tetranin A (**59**) was given to a phenolic compound, while tetranine B (**48**) [42] was given to isoflavonoid, although they were isolated by the same authors. Therefore, the future naming of new isolated compounds from this genus requires careful revision of the previously isolated compounds to avoid any confusion.

The structures of different secondary metabolites are presented in Figure 4, Figure 5, Figure 6, Figure 7, Figure 8, Figure 9, Figure 10, Figure 11, Figure 12, Figure 13, Figure 14, Figure 15, Figure 16 and Figure 17. Meanwhile, a summary of their occurrence in different *Salsola* species is presented in Supplementary Tables S1–S15.

The following section will outline the important isolated and identified compounds in different *Salsola* species, as well as the general procedures of their isolation.

### 4.1. General Procedures for Isolation of Bioactive Compounds from the Genus

Genus *Salsola* is rich in different types of phytoconstituents, for which different techniques are required to isolate their active compounds. Generally, dried plant material is extracted with a suitable organic solvent, such as methanol or aqueous ethanol. Total extract is fractionated with different solvents, *viz.* hexane, chloroform, ethyl acetate and butanol. Hexane fraction is rich in nonpolar constituents, including sterols and triterpenes, which are separated on silica gel columns using an eluting system formed from Hexane:Ethyl acetate with a gradual increase in polarity [43,44,45]. Meanwhile, the chloroform fraction is rich in coumarins, phenolic compounds and flavonoid aglycones. The separation of these compounds is also performed on silica gel columns using chloroform–methanol mixtures with a gradual increase in polarity [46]. Sephadex may be used to purify the isolated compounds using methanol as an eluting agent [46,47,48]. The flavonoid glycosides, as well as saponins, could be detected in ethyl acetate or butanol fractions. These fractions could be treated on Diaion or polyamide columns to remove sugars and obtain flavonoids and their glycosides in a less contaminated form [49,50]. Flavonoid glycosides could be then isolated on normal silica gel using mixtures of chloroform:methanol, with a gradual increase in polarity, or by reverse-phase silica (RP-18) using water:methanol mixtures in isolation [49,50]. Saponins need different treatment, as they were detected in the butanol fraction and could be purified using silica gel columns and chloroform:methanol with a gradual increase in polarity [51]. Alkaloids are usually detected in chloroform or ethyl acetate fractions and separated on silica gel columns using mixtures of chloroform:methanol with a gradual increase in polarity [47,52,53]. Cardinolides are usually detected in chloroform (aglycones) or in butanol (glycosides). Aglycones are separated on silica gel columns using mixtures of chloroform:methanol with gradual increase in polarity; meanwhile, their glycosides are isolated on RP-18 eluted with H_2_O-MeOH [54].

### 4.2. S. baryosma (Schult.) Dandy (Caroxylon imbricata var. imbricatum)

*S. baryosma* has tested positively for alkaloids [55], flavonoids coumarins and sterols [46]. Phytochemical investigation of the chloroform soluble fraction of *S. baryosma* resulted in the isolation of polyoxygenated triterpenes named salsolin A (**142**) and salsolin B (**143**), along with 2α,3β,23,24-tetrahydroxyurs-12-en-28-oic acid (**144**) [44]. In addition, salsolide (**64**), *p*-hydroxyphenylglycol derivative, coumarins as scopoletin (**211**), bergaptol (**218**), daphnoretin (**208**), bergaptol-5-*O*-*β*-d-glucopyranoside (**219**), daphnorin (**209**) and a flavonoid, chrysoeriol-7-*O*-*β*-d-glucopyranoside (**30**), have been isolated from the ethyl acetate soluble fraction of the whole plant [46,56]. Meanwhile, salsolic acid (**140**), an oleane-type triterpene, was isolated from the chloroform fraction of *S. baryosma* [44]. Kaempferol (**18**) and quercetin (**1**) have been isolated from the root, shoot and fruit of *S. baryosma*. Among plant parts, a maximum content of total flavonoids (kaempferol (**18**) and quercetin (**1**)) was observed in fruits, followed by shoot and roots [57].

### 4.3. S. collina Pall.

The herb *S. collina* contains various amino acids, flavonoids, glycosides, steroids, glycoalkaloids and vitamins [58,59]. A previous investigation of the aerial part showed the presence of alkaloids that were isolated and identified as pericampylinone-A (**120**), salsoline A (**114**), *N-trans*-feruloyl-3-*O*-methyldopamine (**102**), salsoline B (**115**), moupinamide (**96**), 2′-hydroxymoupinamide (**97**), 2′-hydroxy-3″-methylmoupinamide (**98**), uracil (**117**), uridine (**118**), *N*-acetyltryptophan (**121**). Glycoalkaloids, salsoline (**110**) and salsolidine (**112**) were also isolated from the aerial parts and extracted with aqueous or aqueous alcohol with an alcohol concentration of 30%, 50% and 70%. The identification of acyl transferases mediating the production of these amino acid phenolic conjugates has yet to be determined considering their abundance among most listed *Salsola* species. It was found that the largest content of alkaloids was extracted with 70% alcohol [58]. It also contained terrestric acid (**119**), anisic acid (**73**), protocatechuic aldehyde (**79**), vanillin (**192**), corchoionoside C (**138**), ferulic acid (**90**), acetyl ferulic acid (**92**), *p*-hydroxycinnamic acid (**88**), *p*-hydroxybenzoic acid (**71**), salicylic acid (**72**), kaempferol (**18**), isorhamnetin (**8**), isorhamnetin-7-*O*-*β*-d-glucopyranoside (**12**), isorhamnetin-3-*O*-*β*-d-glucopyranoside (**9**) and isorhamnetin-3-*O*-*α*-l-arabinopyranosyl(1→6)-*β*-d-glucopyranoside (**16**), selagin (**27**), acanthoside D (**63**), tricin (**26**), tricin-7-*O*-*β*-d-glucopyranoside (**28)**, tricin-4′-*O*-*β*-d-apioside (**29**), 5,2′-dihydroxy-6,7-methylenedioxyisoflavone (**47**), quercetin (**1**), quercetin-3-*O*-*β*-d-glucopyranoside (**3**), quercetin-3-*O*-rutinoside (rutin) (**6**), and narcissin (**13**) [47,52,53,60,61].

Butanol fraction of *S. collina* aerial parts afforded tricin derivatives that were identified as Salcolin A (**23**) was identified as tricin 4′-*O*-(erythro-*β*-guaiacylglyceryl) ether while, Salcolin B (**24**) was identified as tricin 4′-*O*-(threo-*β*-guaiacylglyceryl) ether [62]. Hexane and chloroform fractions of the aqueous ethanolic extract of epigeal part of *S. collina* afforded sterol as *β*-sitosterol (**149**), stigmasterol (**146**), campesterol (**151**), sitostanol (**145**) and their glycoside together with fatty acids as palmitic acid (**180**), oleic acid (**178**), linoleic acid (**170**) and linolenic acid (**172**) [60,63] The major components in the ethyl acetate fraction of *S. collina* were identified using HPLC and LC/MS analysis. Nine compounds were assigned as orsellic acid (**85**), protocatechuic acid (**75**), caffeic acid (**89**), salicylic acid (**72**), vanillic acid (**78)**, syringic acid (**77**), 4-hydroxycinnamic acid (**88**), ferulic acid (**90**) and 4-hydroxybenzoic acid (**71**) [64]. Meanwhile, butanol fraction of seeds of *S. collina* which were exhaustively extracted with ethyl alcohol afforded glycine betaine (**122**) and flavonoids as isorhamnetin (**8**), kaempferol (**18**), quercetin (**1**), isorhamnetin-3-*O*-*β*-d glucopyranoside (**9**), quercetin-3-*O*-*β*-glucopyranoside (**3**), quercetin-3-*O*-rutinoside (rutin) (**6**) [63]. Moreover, different carbohydrates, such as d-glucose and d-fructose, carbohydrate ethers, such as ethyl-*β*-d-glucopyranoside and ethyl-*β*-d-fructopyranoside, and polyhydric alcohols, such as myo-inositol and d-mannitol, were also extracted from the butanol soluble fraction of an ethanolic extract of *S. collina* [65].

### 4.4. S. cyclophylla (Baker) (Synonyme of Caroxylon cyclophyllum (Baker) Akhani and Roalson)

Volatile constituents from *S. cyclophylla* herb were identified by GC and GC/MS and showed thirty-two volatile compounds (98.16%). A total of 34.59% belonged to ketones, aldehydes, and ester, and 27.97% accounted for benzoic acid ester derivatives including mainly benzyl salicylate (**194**) (9.07%). Furthermore, the ketone hexa hydrofarnesyl acetone (**195**) made up 27.14% of the constituents of *S. cyclophylla* volatile oils. In addition, saturated, and unsaturated hydrocarbons were also detected in the volatile constituents. Therefore, benzoic acid ester derivatives, as well as saturated hydrocarbons, are the major constituents of essential oil from *S. cyclophylla* [39]. Benzoate esters were found in *S cyclophylla*, although cinnamate esters have been found in other species. It is now necessary to identify the biochemical pathways involved in the formation of benzoates versus cinnamates.

### 4.5. S. foetida Vest ex Schult. (Synonyme of Suaeda foetida (Vest ex Schult.) Moq.)

A phytochemical study of the whole plant of *S. foetida* lead to isolation of three nitrogenous compounds; *N*-[2′-(3″,4″-dihydroxyphenyl)-2′-hydroxyethyl]-3-(4‴-methoxyphenyl)prop-2-enamide (**99**), *N*-[2′-(3″,4″-dihydroxyphenyl)-2*′*-hydroxyethyl]-3-(3‴,4‴-dimethoxyphenyl)prop-2-enamide (**100**) and *N*-[2*′*-(3″-hydroxy-4″-methoxyphenyl)-2′-hydroxyethyl]3-(4‴-methoxyphenyl)-prop-2-enamide (**101**) [55].

### 4.6. S. grandis Freitag, Vural and Adigüzel

Ethanolic extract of *S. grandis* aerial parts afforded ten flavonoids: isorhamnetin-3-*O*-rutinoside (**13**), quercetin-3-*O*-rutinoside (**6**), quercetin-3-*O*-methyl ether (**2**), tiliroside (**22**), isorhamnetin-3-*O*-glucuronide (**10**), isorhamnetin-3-*O*-glucoside (**9**), quercetin-3-*O*-galactoside (**4**), quercetin-3-*O*-rhamnoside (**5**), quercetin (**1**) and manghaslin (**17**), and two oleanane-type saponins, momordin II b (**127**) and momordin II c (**128**), and one amino acid derivative, *N*-acetyltryptophan (**121**) [49,50].

### 4.7. S. imbricata Forssk. Moq. (Synonyme of Caroxylon imbricatum (Forssk.) Moq.)

This is a tiny shrub that grows to a height of 0.3–1.2 m and is found across Egypt. The Arabic name of *S. imbricata* is “harm”, and it is used as a source of camel food [34]. Chemical investigation of different parts of *S. imbricata* could isolate steroids, triterpenoids, triterpene glycoside, isoflavonoids, flavonoids, anthraquinones, tannins, coumarins, alkaloids, phenolics and sterols [66,67]. Methanol extract of its root afforded 3-*O*-*β*-d-xylopyranosyl-(1→2)-*O*-*β*-d-glucuronopyranosyl-akebonic acid-28-*O*-*β*-D-glucopyranoside (**136**), 3-*O*-*β*-d-xylopyranosyl-(1→2)-*O*-*β*-d-glucuronopyranosyl-29-hydroxyoleanolic acid-28-*O*-*β*-d-glucopyranoside (**137**), pseudoginsenoside RT1 (**129**), and momordin II b (**127**) [68], in addition to nor-triterpene glycoside boussingoside A2 (**135**) [51,69,70]. Ethyl acetate soluble fraction of the alcoholic extract from their roots afforded an alkaloidal phenolic, *N*-*trans*-feruloyltyramine (**103**), in addition to isovanillic acid (**83**), ferulic acid (**90**) and *p*-hydroxy benzoic acid (**71**) [71]. Moreover, Bi-phenylpropanoids, named biphenylsalsonoid A (**62**) and biphenylsalsonoid B (**61**), were also isolated [72].

The flavonol quercitrin (**5**) and the phenolic acid rosmarinic acid (**87**) were isolated from the whole plant of *S. imbricata*. Methanolic extract of their leaves afforded nine phenolic compounds; among them, two compounds were isolated from the butanol fraction, isorhamnetin-3-*O*-*β*-d-glucuronyl(1‴→4″)-*β*-d-glucuronic acid (**14**) and isorhmnetin-3-*O*-*β*-d-diglucuronate dimethyl ester (**15**). Meanwhile, ethyl acetate fraction afforded seven compounds, from which three were identified: isorhamnetin-3-*O*-*β*-d-galactopyranoside (**11**), isorhamnetin-3-*O*-*β*-d-glucopyranoside (**9**) and isorhamnetin (**8**).

Furthermore, HPLC analysis of the hydrolyzed–methanolic extract resulted in the identification and quantification of polyphenols, namely, phenolic acids and flavonoids, using two different wavelengths. At a short wavelength, gallic acid (**76**), protocatechuic acid (**75**), chlorogenic acid (**93**), caffeic acid (**89**), vanillic acid (**78**), ferulic acid (**90**), salicylic acid (**72**) and cinnamic acid (**91**) were the main identified phenolic acids, with a predominancy of *p*-hydroxy cinnamic acid (**88**) (4.251%). Apart from catechol (**58**), two flavonoids, catechin (**52**) and chrysin (**31**), were found; nevertheless, only one non-phenolic compound was identified as benzoic acid (**74**).

On the other hand, at a longer wavelength (*λ* = 330 nm), eight components were identified, among which, seven were flavonoids: quercetin (**1**), hesperidin (**51**), rutin (**6**), naringenin (**49**), hesperetin (**50**) and apigenin (**25**) with major quercitrin (**5**) (12.692%). Rosmarinic acid (**87**) was the only detected phenolic acid [67]. Meanwhile, alcoholic extract from aerial parts of *S. imbricata* yielded two secondary metabolites: salisomide (**124**) and salisoflavan (**46**) [73]. Investigation of the role of rosmarinic acid in that species and the involved biosynthetic pathways can help further agronomic and molecular approaches to improve its yield.

### 4.8. S. inermis Forssk. (Synonyme of Caroxylon inerme (Forssk.) Akhani and Roalson)

Alcoholic extract from *S. inermis* aerial parts afforded 9,12,13-trihydroxydecosan–10,15,19-trienoic acid (**156**); *trans*-*N*-feruloyl tyramine-4‴-*O*-*β*-d-glucopyranoside (**104**); umbelliferone (**210**); scopoletin (**211**); olean-12-en-3,28-diol (**134**); olean-12-en-28-oic acid (**133**); hypogallic acid (**84**); (-) epicatechin (**53**); kempherol (**18**); kaempferol 3-methyl ether (**19**); kaempferol-3-*O*-*β*-glucopyranoside (**20**); quercetin-3-rutinoside (**6**); isorhamnetin-3-*O*-*β*-glucopyranoside (**9**); stigmasterol-3-*β*-*O*-d-glucopyranoside (**147**); *β*-sitosterol (**149**); stigmasterol (**146**) and stigmastanol (sitostanol) (**145**) [35].

### 4.9. S. kali L. (S. spinosa Lam.)

Aerial parts of *Salsola kali* L. contains tetrahydroisoquinoline alkaloids; salsoline (**110**), salsolidine (**112**), *N*-methylisosalsoline (**111**) and carnegine (**113**) which were also separated from the aerial parts of *S. soda* L., *S. oppositifolia* and *S. ruthenica* methanol extract [74].

Its aerial parts contained some fatty acids, such as linolenic (**172**), oleic (**178**), arachidonic (**162**), palmitic (**180**) and stearic (**182**). Moreover, its aerial parts afforded sterols such as *β*-sitosterol (**149**), *β*-sitosterol-3-*O*-glucoside (**150**), sitostanol (**145**), stigmasterol (**146**) and avenasterol (**148**), which were also found in *S. tetrandra*, *S. rigida* and *S. longifolia* [75,76]. Additionally, triterpenes such as lupeol (**139**), and ursolic acid (**141**) were found in the whole plant [45].

Moreover, kempferol (**18**), isorhamnetin-3-*O*-glucoside (**9**), isorhamnetin-3-*O*-rutinoside (narcissin) (**13**), rhamnetin (**7**), quercetin (**1**), quercetin-3-glucoside (**3**), quercetin-3-rhamnoside (**5**) and quercetin-3-rutinoside (rutin) (**6**) were also identified in *S. kali* [35,38]. In addition, caffeic (**89**), ferulic (**90**), chlorogenic (**93**), isochlorogenic (**95**) and neo-chlorogenic (**94**) were the major phenolic acids identified in leaves of *S. kali* L. [13,38].

Moreover, the aerial parts and roots of *S. kali* afforded phenolic acids that were free or liberated from their sugar after hydrolysis. The phenolic acids were identified as protocatechuic (**75**), caffeic (**89**), gentisic (**82**), *p-*hydroxy cinnamic (**88**), *p*-hydroxybenzoic (**71**), *p*-hydroxyphenylacetic (**86**), syringic (**77**), vanillic (**78**), ferulic (**90**), *α* and *β*-resorcylic (**80, 81**). Gentisic (**82**), *p*-hydroxyphenylacetic (**86**) and *β*-resorcylic (**81**) [12]. Detailed phytochemical profiling, in parallel with gene expression, can help establish different biosynthetic pathways in different organs. Moreover, hypogallic acid (**84**) and gallic acid (**76**), the precursor of hydrolysable tannins, were found in their aerial parts. (-)Epicatchin (**53**), which is the condensed tannins precursor, was found in most of the *Salsola* species, except *S. kali* and *S. tetragona* [35].

### 4.10. S. komarovii Iljin

Methanol extract of *S. komarovii* aerial parts afforded five lignan glycosides: lariciresinol-9′-*O*-*β*-d-glucopyranoside (**198**), alangilignoside C (**199**), conicaoside (**200**), (+)-lyoniresinol 9′-*O*-*β*-d-glucopyranoside (**196**) and (*8S,8′R,7′R*)-9′-[(*β*-glucopyranosyl)oxy]lyoniresinol (**197**); seven megastigmane glycosides, identified as blumenyl B *β*-d-glucopyranoside (**206**), blumenyl A *β*-d-glucopyranoside (**203**), staphylionoside D (**202**), icariside B_2_ (**201**), (*6R,9S*)-3-oxo-*α*-ionol *β*-d-glucopyranoside (**204**), 3-oxo-*α*-ionol 9-*O*-*β*-d-apiofuranosyl-(1→6)-*β*-d-glucopyranoside (**205**) and blumenol B 9-*O*-*β*-d-apiofuranosyl-(1→6)-*β*-d-glucopyranoside (**207**); and seven phenolic compounds, determined as benzyl 6-*O*-*β*-d-apiofuranosyl-*β*-d-glucopyranoside (**57**), canthoside C (**67**), tachioside (**69**), isotachioside (**70**), biophenol 2 (**54**), 2-(3,4-dihydroxy)-phenyl-ethyl-*β*-d-glucopyranoside (**55**) and cuneataside C (**56)** [41]. Moreover, seven flavonoids, rutin (**6**), isoquercitrin (**3**), kaempferol-3-*O*-rutinoside (**21**), isorhamnetin-3-*O*-rutinoside (**13**), kaempferol 3-*O*-glucoside (astragalin) (**20**), isorhamnetin-3-*O*-glucoside (**9**), isorhamnetin (**8**) and two phenolic amides, identified as *N*-*trans*-feruloyl tyramine (**103**) and *N*-*trans*-feruloyl-3-*O*-methyldopamine (**102**), were identified from the aerial parts of the ethyl acetate fraction of *S. komarovii* [77].

### 4.11. S. laricifolia Litv. ex Drobow

Ethanol extract of *S. laricifolia* epigeal parts, which is collected in the fruit-bearing period from SouthGobi arimak, Mongolia, contained coumarins that were identified as fraxidin (**213**), isofraxidin (**214**), fraxetin (**215**), fraxidin-8-*O*-*β*-d-glucopyranoside (**217**), isofraxidin-7-*O*-*β*-d-glucopyranoside (calycanthoside) (**216**) from the CHCl_3_ fraction and scopoletin-7-*O*-*β*-glucopyranoside (**212**) from EtOAc and BuOH fractions [29].

### 4.12. S. longifolia Forssk.

*S. longifolia* stem was reported to contain kempferol (**18**), quercetin (**1**), quercetin-3-rhamnoside (**5**), gentisic acid (**82**), protocatchuic acid (**75**) and (-)epicatechin (**53**) [35].

### 4.13. S. micranthera Botsch. (Synonym of Caroxylon micrantherum (Botsch.) Sukhor.)

Salsolosides C (**130**), D (**131**), and E (**132**) are triterpene glycosides isolated from the aerial part of *S. micranthera* [78,79].

### 4.14. S. oppositifolia Pall.

Isorhamnetin-3-*O*-glucoside (**9**) and isorhamnetin-3-*O*-rutinoside (**13**) flavonols were isolated from ethyl acetate fraction of aerial parts of *S. oppositifolia*. Meanwhile, methyl palmitate (**173**), palmitic acid (**180**), methyl stearate (**176**), *β*-sitosterol (**149**), methyl linolenate (**171**), phytol (**226**), 2-monolinolenin (**152**) were the major constituents isolated from *n*-hexane fraction while linoleic acid (**170**), 2-monolinolenin (**152**), palmitic acid (**180**), methyl linolenate (**171**) and methyl linoleate (**159**) were identified from the CH_2_Cl_2_ fraction using GC-MS. In addition, GC-MS analysis of the diethyl ether fraction revealed the presence of salsoline (**110**) and salsolidine (**112**) alkaloids [80].

### 4.15. S. soda L. (Synonym of Soda inermis Fourr.)

Chemical investigations of wild and cultivated *S. soda* revealed the presence of four flavonoids: rutin (**6**), quercetin-3-*O*-glucouronopyranoside (**3**), isorhamnetin-3-*O*-rutinoside (**13**), and isorhamnetin-3-*O*-glucuronopyranoside (**10**). Furthermore, a saponin, momordin II c (**128**), was identified. Even at the young twigs stage, when it is used as food, cultivated *S. soda* produced a significant number of secondary metabolites. Both flavonoids and saponins were found in varying amounts in the two types, according to the LC-MS quantitative analysis [81].

### 4.16. S. somalensis N.E.Br.

Roots of *S. somalensis* afforded twelve isoflavones, 5,3′-dihydroxy-7,8,2′-trimethoxyisoflavone (**32**), 5,3′-dihydroxy-2′-methoxy-6,7-methylenedioxyisoflavone (**33**), 5,3′-dihydroxy-6,7,8,2′-tetramethoxyisoflavone (**34**), 5,3′-dihydroxy-6,7,2′-trimethoxyisoflavone (**35**), 5,8,3′-trihydroxy-7,2′-dimethoxyisoflavone (**36**), 8,3′-dihydroxy-5,7,2′-trimethoxyisoflavone (**37**), 5,6,3′-trihydroxy-7,2′dimethoxyisoflavone (**38**), 6,7,3′-trihydroxy5,2′- dimethoxyisoflavone (**39**), 5,8,3′-trihydroxy-2′-methoxy-6,7-methylendioxyisoflavone, or 5,6,3′-trihydroxy-2′-methoxy-7,8-methylenedioxy isoflavone (**40**), 3′-hydroxy-5,6,7,2′-tetramethoxyisoflavone (**41**), 7,3′-dihydroxy-5,6,2′-trimethoxyisoflavone (**42**) and 6,3′-dihydroxy-5,7,2′-trimethoxyisoflavone (**43**) besides two more compounds named as 5,7,8,2′,3′-pentamethoxyisoflavone (**44**) and 5,2′,3′-trimethoxy-6,7-methylendioxyisoflavone (**45**) [24,25]. While isoflavones are restricted in few plant families mostly legumes, their ecological role in *Salsola* has yet to be determined.

### 4.17. S. tetragona Delile (Synonym of Caroxylon tetragonum (Delile) Moq.)

The aerial parts of *S. tetragona* afforded five cardenolides: salsotetragonin (**220**), uzarigenin (**221**), desglucouzarin (**222**), 12-dehydroxy-ghalakinoside (**223**) and calactin (**224**); three flavonoids: quercetin-3-rutinoside (**6**), kaempferol-3-*O*-*β*-d-glucopyranoside (**20**) and quercetin-3-*O*-*β*-d-glucopyranoside (**3**); and four phenolic compounds: vanillic acid (**78**), protocatchuic acid (**75**), canthoside C (**67**) and canthoside D (**68**); in addition to two fatty acids: oleic acid (**178**) and 2,3-dihydroxypropylpalmitate (**153**) [35,54]. Whether cardenolides exist in other species has yet to be confirmed by the profiling of many other species for comparison.

### 4.18. S. tetrandra Forssk. (Synonym of Caroxylon tetrandrum (Forssk.) Akhani and Roalson)

Coumarins, saponins, alkaloids, terpenes, and steroids were detected in aqueous ethanol extract from the aerial portions of *S. tetrandra* [26]. The metabolite profile of the methanol extract of aerial parts and root of *S. tetrandra* detected a total of 29 metabolites, from which only 24 were identified using ultra-performance liquid chromatography coupled to mass spectrometry (UPLC-MS) and nuclear magnetic resonance (NMR). The classification of detected metabolites was assessed using principal component analysis (PCA). Under optimized conditions, the discovered metabolites belonged to distinct classes, including five hydroxycinnamic acid conjugates of norepinephrine and tyramine as *N*-caffeoyl tyramine (**106**), *N*-*trans*-feruloyl tyramine (**103**), *N*-(3′,4′-dimethoxy-cinnamoyl)-norepinephrine (**108**), *N*-(4′-methoxy-cinnamoyl)-norepinephrine (**109**) and *N*-feruloyl-3‴-methoxy tyramine (**107**); six flavonoids with a high abundance of kaempferol derivatives, identified as kaempferol trihexoside, kaempferol pentosyl dihexoside, kaempferol-*O*-rhamnosyl dihexoside, rutin (**6**), isorhamnetin-3-*O*-rutinoside (**13**) and isorhamnetin-3-*O*-glucopyranoside (**9**); eight fatty acid derivatives, identified as 9,12,13-trihydroxy octadeca-7-enoic acid (**155**), trihydroxy octadecadienoic acid (**183**), hydroxy octadecatrienoic acid (**165**), hydroxy octadecadienoic acid (**166**), octadecenoic acid (oleic acid) (**178**), octadecatrienoic acid (linolenic acid) (**172**), octadecadienoic acid (linoleic acid) (**170**), palmitic acid (**180**), and a nitrogenous compound identified as salsoline A (**114**). The aerial parts were higher in flavonoids, whereas the roots were higher in hydroxycinnamic acid conjugates [26]. Few studies have reported on the application of chemometrics for classification and or differentiation between the different *Salsola* species, and this should be considered in the future, and from larger specimens, to help identify which species presents the best source of a certain class or the best identification of markers.

On the other hand, different compounds are present in the unsaponifiable matter from petroleum ether extract, including different compounds, such as tridecanamine (**126**), 2,7-dimethyl-1-octanol (**228**), isohexyl-2-pentylester sulfurous acid (**231**), 3,9-diethyl-6-tridecanol (**227**), methyl palmitate (**173**), 8-hexadecynoic acid (stearolic acid) (**154**), 9,12-octadecadienoic(*Z,Z*), methyl ester (methyl linoleate) (**159**), octadecanoic acid, 2,3-dihydroxypropyl ester (monostearin) (**179**), myristic acid methyl ester (**184**), long chain fatty acids methyl esters, as lauric acid (**167**), myristic acid (**175**), palmitic acid (**180**), palmitoleic acid (**181**), heptadecanoic (margaric) acid (**174**), *cis*-10- heptadecanoic acid (**186**), stearic acid (**182**), oleic acid (**178**), nonadecanoic acid (**169**), linoleic acid (**170**), icosanoic (arachidic) acid (**161**), linolenic acid (**172**), 11- eicosenoic acid (**160**), docosanoic (behenic) acid (**163**), tricosanoic acid (**185**), tetracosanoic (lignoceric) acid (**168**), hexacosanoic acid (**164**) and octacosanoic acid (**177**). Saturated fatty acid content reached 43.16%, while unsaturated fatty acid content with 56.84% (with a predominancy of polyunsaturated FA, at 48.59%, while monounsaturated fats comprised 8.25%) [82]. Furthermore, given the limited phytochemical research into this species that has been published, a coumarinolignan, estrone, cholesterol and three bases, identified as triacetonamine (**125**), betaine (**122**) [83] and methyl carbamate (**123**) [75], have been identified and were also detected in *S. kali*, *S. longifolia* and *S. rigida* [83].

The aerial parts of *S. tetrandra* afforded norisoprenoid; 3-*β*-hydroxy-5*α*,6*α*-epoxy-*β*-ionone-2-*α*-*O*-*β*-d-glucopyranoside (**230**), long-chain hydroxyl fatty acids 9,12,13-trihydroxyoctadeca-10(*E*),15(Z)-dienoic acid (**157**) and 9,12,13-trihydroxyoctadeca-10(*E*)-dienoic acid (**158**) in addition to 3,4,5-trimethoxyphenyl-*β*-d-glucopyranoside (**66**), 9-hydroxylinaloyl glucoside (**189**), taxiphyllin (**229**), *N*-*trans* feruloyltyramine (**103**) and *S-*(-)-*trans*-*N*-feruloyloctopamine (**105**) [70].

Tetranin A (**59**) (bibenzyl derivative) and isoflavonoid; tetranin B (**48**) were isolated from the roots of *S. tetrandra* [42]. Flavonoids, quercetin (**1**), rutin (**6**), kempherol (**18**) and other phenolic compounds as hypogallic acid (**84**), phloroglucin (**65**) and (-) epicatechin (**53**), were isolated from *S. tetrandra* stem [35].

### 4.19. S. tomentosa (Moq.) Spach

Phenolic components (tannins, flavonoids, and total phenols), and saponins were detected as major constituents of the aerial parts of *S. tomentosa* collected from Qum province in Iran. Methanol extraction, either by soxhelt or maceration, provided the highest concentration of total phenolic and flavonoid [84].

### 4.20. S. vermiculata L. (Synonym of Caroxylon vermiculatum (L.) Akhani and Roalson)

This is an annual plant with a wide distribution range in Southwest Asia [85]. The metabolite profile of *S. vermiculata* reveal a total of 28 metabolites, only 24 of which were identified in the methanol extract of aerial portions and root using ultra-performance liquid chromatography coupled to mass spectrometry (UPLC-MS) and nuclear magnetic resonance (NMR). The classification of detected constituents was performed using principal component analysis (PCA). Under optimized conditions, the identified metabolites belonged to various classes, including five hydroxycinnamic acid conjugates of norepinephrine and tyramine, namely, *N*-caffeoyl tyramine (**106**), *N*-trans-feruloyl tyramine (**103**), *N*-(3′,4′-dimethoxy-cinnamoyl)-norepinephrine (**108**), *N*-(4′-methoxy-cinnamoyl)-norepinephrine (**109**) and *N*-feruloyl-3‴-methoxy tyramine (**107**); six flavonoids, namely, kaempferol trihexoside, kaempferol pentosyl dihexoside, kaempferol-*O*-rhamnosyl dihexoside, rutin (**6**), isorhamnetin-3-*O*-rutinoside (**13**) and isorhamnetin-3-*O*-glucopyranoside (**9**); eight fatty acid derivatives, namely, 9,12,13-trihydroxy octadeca-7-enoic acid (**155**), trihydroxy octadecadienoic acid (**183**), hydroxy octadecatrienoic acid (**165**), hydroxy octadecadienoic acid (**166**), oleic acid (**178**), linolenic acid (**172**), linoleic acid (**170**), palmitic acid (**180**); and two nitrogenous compounds, namely, salsoline A (**114**) and *N*-(4-methylpentanoyl) tyramine (**116**). Hydroxycinnamic acid conjugates were plentiful in the roots, whereas areal parts were rich in flavonoids, with quercetin derivatives being the most common flavonoids [26].

The volatile fractions produced by hydrodistillation of *S. vermiculata* leaves, stems, and roots were chemically analyzed, and forty-four compounds were identified, belonging to several chemical classes. Twenty-eight constituents made up 95.9% of the total constituents in the volatile fraction of leaves. The major compounds of this fraction were carvone (**187**) (52.2%), cumin aldehyde (**193**) (6%), *β*-caryophllene (**191**) (5.8%) and linalool (**188**) (7.1%). Meanwhile, sixteen compounds were identified, representing 98% the of volatile fractions from the stem. The main identified compounds were carvone (**187**) (53%), limonene (**190**) (17.4%), linalool (**188**) (11.3%) and *β*-caryophllene (**191**) (7.5%). Thirty-three constituents, amounting to 94% of the total, were identified from volatile constituents of the root. Most compounds were carvone (**187**) (49.9%), *β*-caryophllene (**191**) (8.5%), linalool **(188**) (8.2%) and cumin aldehyde (**193**) (4.4%). Oxygenated monoterpenes are the dominant class of volatile fractions present in S*. vermiculate.* Carvone (**187**) is the main major component of this class [86]. Few studies have been presented on volatile composition in *Salsola* species, and these should be compared to the composition reported for *S. vermiculate* in the future.

### 4.21. S. villosa Schult. (Synonym of Caroxylon villosum (Schult.) Akhani and Roalson)

The phytochemical screening of the 95% ethanol extract of the whole plant of *S. villosa* revealed the presence of alkaloids, saponins, tannins, flavonoids, sterols/terpenes and coumarins [87]. Previous work led to the isolation of secondary cyclic alcohol, salsolanol (**225**) and biphenylsalsinol (**60**) from the chloroform fraction of the aerial parts of *S. villosa* [88]. Compared to other reports on aerial parts’ chemical composition, few studies have looked at root organs in most *Salsola* species.

### 4.22. S. volkensii Schweinf. and Asch.

Quercetin (**1**), quercetin-3-glucoside (**3**), quercetin-3-rutinoside (**6**), hypogallic acid (**84**), phloroglucin (**65**) and (-) epicatechin (**53**) was isolated from the stem of *S. volkensii* [35].

## 5. Overview of the Benefits, Uses and Medicinal Properties of *Salsola* Genus

**Figure 18:**
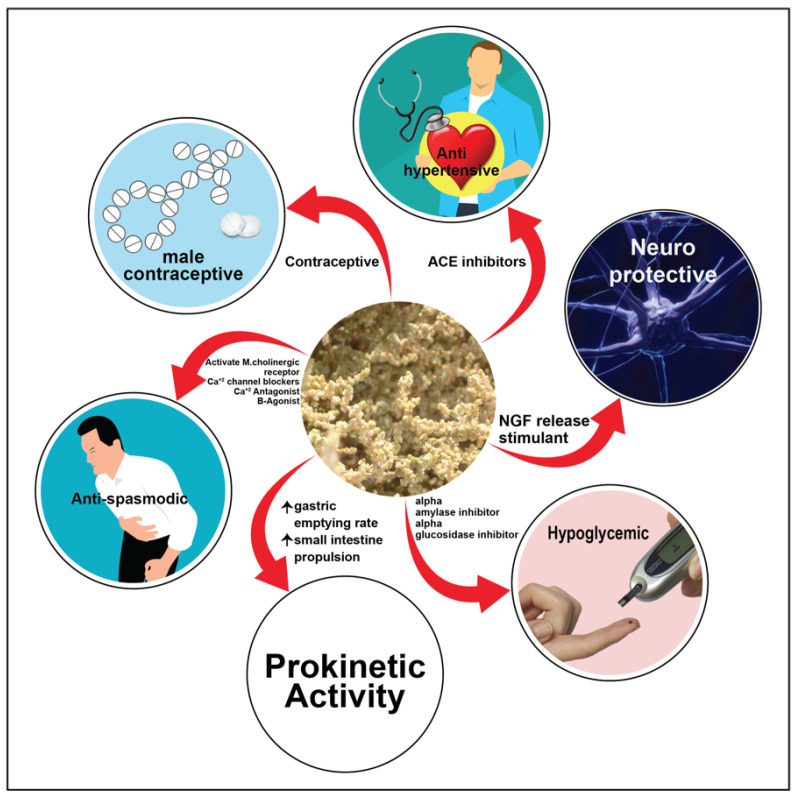
Some important biological activities of genus *Salsola* and their mechanism.

**Figure 19:**
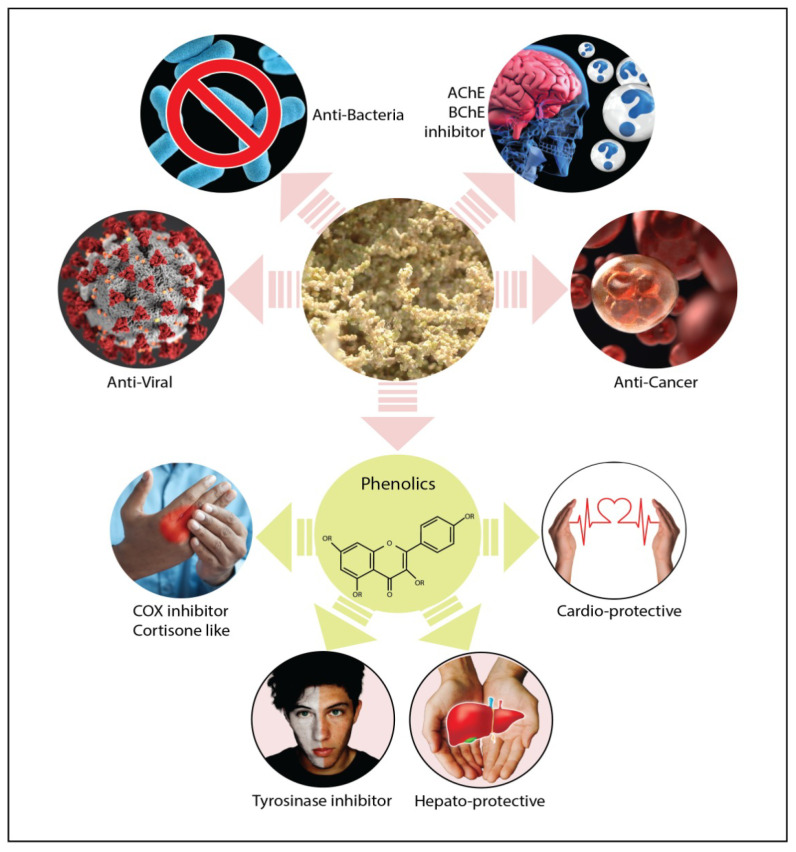
Important biological activities of genus *Salsola*.

There have only been a few chemical and biological studies of *Salsola* genus. Halophytic plants have been used for medicinal purposes due to the presence of health-promoting bioactive compounds [89]. In this regard, members in *Salsola* genus have a significant therapeutic value (Figure 18 and Figure 19). *Salsola* species have a variety of constituents, with a wide range of biological activities, and have been reported to be utilized in folk medicine all throughout the world, according to the literature. In the following sections, we will go through the different medicinal uses of this genus. The authors will outline the benefits and medicinal uses of different species in the genus *Salsola*.

### 5.1. Anti-Inflammatory, Analgesic and Anti-Nociceptive Activity

The incidence of inflammatory diseases is becoming common in almost all countries around the world. Despite their well-known side effects, non-steroidal anti-inflammatory drugs are most commonly used to relieve inflammatory pain [90]. Natural products and traditional medicines, as alternatives to these drugs, offer great hope in the development of efficient agents for the treatment of inflammatory diseases [91]. In this regard, total methanol extract, together with petroleum ether, chloroform, and ethyl acetate fractions of *S. kali,* were investigated for their anti-inflammatory activity using rat paw edema test. The petroleum ether fraction demonstrated the highest activity (60%). Meanwhile, the chloroform, ethyl acetate fractions and methanol extract led to a 35.0%, 20% and 40% reduction in rat-paw, respectively, relative to indomethacin [45]. The significant anti-inflammatory activity produced by the petroleum ether fraction was attributed to its sterols’ contents lupeol (**139**), ursolic acid (**141**), *β-*sitosterol (**149**) and *β*-sitosterol-3-*O*-glucoside (**150**), which were detected in petroleum ether extract of *S. kali*. Moreover, these compounds were proven to be anti-inflammatory by different mechanisms [92,93,94]. Moreover, phenolic acid, ferulic (**90**), which was also identified in *S. kali,* is known for its strong anti-inflammatory activity [12,95].

The total aqueous methanol extract of *S. imbricata* leaves and the six isolated phenolic compounds, isorhamnetin-3-*O*-*β*-d-glucuronyl (1‴→4″)-*β*-d-glucuronic acid (**14**), isorhamnetin-3-*O*-*β*-d-diglucuronate dimethyl ester (**15**), isorhamnetin-3-*O*-*β*-d-galactopyranoside (**11**), isorhamnetin-3-*O*-*β*-d-glucopyranoside (**9**), isorhamnetin (**8**), *N*-*trans*-feruloyltyramine (**103**), distinctly showed in vitro anti-inflammatory activities, with no toxicity, in Raw murine macrophages cells (RAW 264.7) using a nitric oxide assay at a concentration level of 100 µg/mL for all samples. It is noteworthy that isorhamnetin-3-*O*-*β*-d-glucopyranoside (**9**) showed the highest anti-inflammatory activity [71]. An in vivo model should be used in further studies to make the results more conclusive.

COX and other mediators implicated in the pathophysiology of pain alleviation, as well as anti-nociceptive activity, are inhibited by a hydroalcoholic extract from the aerial portions of *S. inermis* [96].

Using carrageenan-induced paw edema and *p*-benzoquinone-induced nociception models, the anti-inflammatory and anti-nociceptive effects of the ethanol extract and BuOH fraction of *S. grandis*, as well as their major constituents, were examined in vivo on male Swiss albino mice. The inhibitory effect of the BuOH fraction on carrageenan-induced paw edema was 27.8–32.9%. On the other hand, a 37.6% inhibition was detected in the *p*-benzoquinone-induced nociception model. Tiliroside (**22**) and quercetin-3-*O*-galactoside (**4**) were shown to have the most powerful inhibitory effects in the employed models, according to the findings [49].

*S. komarovii* ethanol extract exhibited anti-inflammatory effects by significantly decreasing lipopolysaccharide (LPS)-induced interleukin IL-6 production, such as hydrocortisone. This worked by a different mechanism to glucocorticoids’ induction, which is the main side effect of gluococorticoids [97].

In addition, the aqueous-ethanolic extract of the aerial parts of *S. cyclophylla* exhibited strong analgesic activity in mice in a hot plate model of pain induction, as well as a carrageenan-induced paw edema model. The activity was attributed to the high phenolic contents of the plant [36].

### 5.2. Antibacterial Activity

Salsoline A (**114**), an alkaloid isolated from *S. collina*, as well as ferulic acid (**90**), a phenolic acid identified in *S. kali*, showed appreciable anti-bacterial activity [95,98,99]. The antibacterial activity of the methanol extract of *S. kali* aerial parts was evaluated using the agar-well diffusion method against seven pathogenic bacterial strains at a concentration of 0.5 μg/mL. The highest activity was against *Staphylococcus aureus*, *Streptococcus mutans*, *Bacillus subtilis* and *Streptococcus pneumoniae*, while moderate bactericidal activity was shown against *Pseudomonas aeruginosa*. The growth of *Escherichia coli* and *Sarcina lutae* was inhibited. Pure methanol was used as a negative control, while Ampicillin, Amoxicillin, Levofloxin, Tetracycline, Vancomycin, Ciprofloxacin, and Penicillin were positive controls [100].

The in vitro anti-bacterial activity of the ethyl acetate extract from the roots of *S. imbricata* and the two biphenylpropanoids A (**62**) and B (**61**) was evaluated by the minimum inhibitory concentration (MIC) method. The two compounds had a similar effectiveness against the tested bacteria, with MIC values ranging from 16 to 64 µg/mL. On the other hand, biphenylsalsonoid B (**61**) showed higher potency than biphenylsalsonoid A (**62**) against *M. luteus* [72].

Taxiphyllin (**229**) and *S*-(−)-*trans*-*N*-feruloyloctopamine (**105**) isolated from *S. tetrandra* displayed mild anti-bacterial activity against *Staphylococcus aureus* at a concentration of 200 µg/mL, with a minimal bactericidal concentration (500 and 600 µg/mL, respectively) [70].

It was found that 95% ethanol extract of the whole plant of *S. villosa*, which contains a high concentration of alkaloid and flavonoid, showed a wide spectrum of anti-microbial activity at different concentrations against *S. aureus* and *P. aeruginosa* using the agar diffusion method and antibiotics discs of Streptomycin and Chloramphenicol as positive controls [87]. Different fractions of *S. villosa* revealed different degrees of anti-microbial activity against gram-positive and -negative micro-organisms [101]. Meanwhile, Oueslati and Al-Ghamdi et al., 2015, stated that salsolanol (**225**) and biphenylsalsinol (**60**) from *S. villosa* exhibited anti-bacterial activities. The highest anti-microbial effect was observed for biphenylsalsinol (**60**) [88].

The anti-microbial activity of extracts prepared from different organs of *S. vermiculate* (10 mg/mL) was evaluated using the microdilution technique to determine the (MIC). *E. faecalis* and *S. aureus* were the most affected by *S. vermiculate* extracts (MICs 0.28 to 4.16 mg/mL). Ethanol extract of the root was the most effective on *S. aureus*, while *E. coli* and *P. aeruginosa* were the most resistant bacteria. The antibacterial activity was referred to as carvone (**187**) [102]. It has the ability to destabilize the phospholipid bilayer, interact with enzymes and proteins in the membrane, and reduce pH gradient across the membrane [103].

The agar diffusion method was used to perform the antimicrobial assay of *S. cyclophylla*. Positive control drug disc 10 µg/mL Amoxicillin and Gentamycin, inhibition zone diameter (IZD) and a broth micro-dilution test were chosen to determine the MIC for selected microorganisms. This had no effect on *Staphylococcus epidermidis*, but was effective against *Staphylococcus aureus* and *Streptococcus pyogenes* with 16 and 11 mm IZD, and an MIC equal to 45 and 72 mg/mL, respectively. Furthermore, it showed activity against *Pseudomonas aeruginosa* Gram-negative strain with 11 mm IZD and 75 mg/mL MIC, respectively. In contrast, it showed 10 mm IZD with an MIC equal to 79 mg/mL against *E. coli*. As a result, potent anti-microbial activity was proven, which is remarkable, as this herb is a common camel feed [39]. It should be noted that most results for extracts and or compounds assessed for antimicrobial assays were based on in vitro or agar diffusion assays, with no animal models tested to confirm efficacy. These studies should now follow.

### 5.3. Anti-Viral Activity

Salsoline A (**114**), an alkaloid in *S. collina*, showed moderate anti-viral activity against influenza virus A and B [98]. The activity was assessed by infection of Madin-Darby canine kidney (MDCK) cell monolayers with influenza virus A or B using ribavirin as a standard antiviral agent. Salsoline A (**114**), showed antiviral activity against influenza virus A with IC_50_ 56.8 µg/mL [98].

### 5.4. Anti-Fungal Activity

The petroleum ether fraction of the whole plant of *S. kali* exhibited a significant in vitro anti-fungal activity against *Rhizoctonea solani* and *Nattrassi mangifera* (21.1 mm and 25.3 mm, respectively) using the agar disc diffusion assay [104]. Mahasneh et al. (1996) studied the anti-fungal activity of the whole aerial parts of the butanol extract of *S. villosa* which showed significant anti-fungal activity (13–14 mm inhibition zones) against *Candida albicans* and *Fusarium oxysporum*, with comparable results to the anti-fungal Miconazole nitrate [101].

The anti-fungal activity of *S. vermiculate* leaf, root, and stem extracts (100 mg/mL) was tested against three pathogenic *Candida* species; *C. glabrata*, *C. krusei* and *C. parapsilosis* using the diffusion method in a solid medium (Sabouraud Chloramphenicol). The results showed that the activity varied according to the pathogen and the plant extract. It also appears that these activities were weak with inhibition zone diameters ranging from 6.5 to 9.5 mm. The butanol fraction of root methanol extract was the most active on *C. parapsilosis* (φIZ = 9.5 mm). The richness of *S. vermiculate* leaves, stems and roots volatile fractions in carvone (**187**) (52.2%, 53% and 49.9%, respectively) could explain its anti-fungal activity [86].

*S. cyclophylla* volatile oil demonstrated a powerful effect against *C. albicans* fungus compared with Clotrimazole standard, with an inhibition zone of 16 mm IZD and 14.5 mg/mL MIC, respectively [39]. Terrestric acid (**119**) from *S. collina* showed positive anti-fungal activity when evaluated by the standard broth micro-dilution method of the NCCLS [47].

### 5.5. Anti-Oxidant, Hepato-Protective and Cardio-Protective Activity

Active polymers such as free radicals (reactive oxygen species or reactive nitrogen species) are overproduced or eliminated too slowly under oxidative stress. A variety of chronic disorders, such as diabetes mellitus (DM) and Alzheimer’s disease, are linked to an oxidation–antioxidation imbalance [105,106,107]. As long as the body maintains a dynamic balance between oxidation and anti-oxidation, excess ROS and RNS can rapidly be removed from the body. Cellular damage occurs as a result of overproduction of RNS and ROS, resulting in damage to all cellular components, including DNA, proteins, and lipids [108], which causes disordered cell function and metabolism. Excess ROS and RNS have been reported to be eliminated by natural antioxidants, as well as preventing free radicals from oxidizing and harming cells.

*Salsola* is an important halophytic genera of the family Amaranthaceae and is considered as a genera of plants containing anti-oxidants compounds with low caloric composition [4]. It has been reported that the ethanol extract of *S. collina* has anti-oxidant activity through its DPPH radical scavenging capacity [109]. Ethyl acetate extract of *S. collina* alleviates diabetic gastroparesis (DGP), possibly by promoting gastric emptying in DGP Male Sprague-Dawley rats, due to its oxidative stress inhibition ability, and increasing the number of gastric neurons, combined with its hypoglycemic and lipid-lowering effects [64].

Polyoxygenated triterpenes salsolin A (**142**) and B (**143**), together with 2*α*,3*β*,23,24-tetrahydroxyurs-12-en-28-oic acid (**144**), have been reported to possess significant anti-oxidant activity in the chloroform soluble subfraction of *S. baryosma* [44]. Moreover, the EtOAc fraction of the whole plant gave 73% anti-oxidant activity, whilst other fractions (ethanol 80%, *n*-hexane and *n*-BuOH) had an anti-oxidant activity below 57%, which was determined using the DPPH radical scavenging method [48].

Biphenylsalsonoid A (**62**) and B (**61**), which was isolated from the roots of *S. imbricata*, showed a moderate activity towards DPPH, with IC_50_ values of 86.5 ±1.3 and 122.3 ± 1.4 µg/mL, respectively, and ABTS with IC_50_ values of 95.1 ± 1.5 and 137.7 ± 1.2 µg/mL, respectively. Biphenylsalsonoid A (**62**) had a relatively higher activity due to the presence of two phenol groups [72]. Quercitrin (**5**) and rosmarinic acid (**87**), both isolated from *S. imbricata*, have been shown to protect against CCl_4_-induced hepatotoxicity and have a high anti-oxidant potential [110].

The in vitro DPPH radical scavenging activity of the methanol extract of the aerial parts of *S. tetrandra* exhibited a strong anti-oxidant activity, with an IC_50_ of 24.98 µg/mL, comparable with ascorbic acid standard (24.7 µg/mL). This finding agrees with the enrichment of the extract with polyphenols, particularly flavonoids [26]. Tetranins A (**59**) (bibenzyl derivative) and B (**48**) (isoflavonoid) were isolated from the EtOAc extract of *S. tetrandra* roots. They demonstrated a significant anti-oxidant effect in DPPH free-radical scavenging activity and ABTS assays. In the DPPH assay, tetranin A (**59**) possessed a higher anti-oxidative capability than tetranin B (**48**), with an IC_50_ of 0.17 mM and 1.09 mM, respectively. In the ABTS assay, tetranin A (**59**) had slightly lower anti-oxidant effects than tetranin B (**48**) with a Trolox-equivalent anti-oxidant capacity (TEAC) of 2.39 mM and 2.06 mM, respectively [42].

The hydroalcoholic extract from the aerial parts of *S. inermis* exhibited anti-oxidant activity [96]. Methanol and acetone extract of the aerial parts of *S. tomentosa* showed good in vitro anti-oxidant activity using the DPPH and *β*-carotene bleaching methods [84].

The qualitative measurement of anti-oxidant activity using a DPPH spraying reagent revealed that *S. cyclophylla* essential oils exhibit some anti-oxidant activity, as fading purple color spots appeared as positive anti-oxidant activity. The scavenging effect of essential oils was 32% when compared with the standard quercetin and Trolox. The anti-oxidant activity may be attributed to the presence of a noticeable proportion of benzoic acid ester derivatives (27.97%) and ketone hexahydrofarnesyl acetone (27.14%) [39].

The ferulic acid (**90**) identified in *S. kali* is known for its strong anti-oxidant activity [12]. It decreases the synthesis of cholesterol and lipids levels and protects against coronary disease [95]. Pretreatment with aqueous extract of *S. kali* (200 mg/kg orally) had a potential anti-oxidant activity, which ameliorated adriamycin (ADR)-induced cardiotoxicity in male Swiss albino mice. These protective mechanisms may be caused by inhibiting lipid peroxidation (LPO) and enhancing anti-oxidant status in the heart [111].

Phenolic compounds isolated from *S. baryosma* were identified as *N*-[2′-(3″,4″-dihydroxyphenyl)-2′-hydroxyethyl]-3-(4‴-methoxyphenyl)prop-2 enamide (**99**), *N*-[2′-(3″,4″-dihydroxyphenyl)-2′-hydroxyethyl]-3-(3‴,4‴-dimethoxyphenyl)prop-2-enamide (**100**) and *N*-[2′-(3″-hydroxy-4″-methoxyphenyl)-2′-hydroxyethyl]3-(4‴-methoxyphenyl)-prop-2-enamide (**101**), and exhibited moderate anti-oxidant activity using a DPPH radical scavenging assay with IC_50_ 383, 427 and 378 µM, respectively. The anti-oxidant potentials of test samples were compared with 3-(*tert-butyl*)-4-hydroxyanisol and propylgallate as a positive control [55].

The phenolic anti-oxidant constituents in the aerial parts of *S. komarovii* extract were determined using the online, HPLC-coupled, ABTS^+^-based assay (HPLC)-ABTS^+^, while HPLC with electrospray ionization-mass spectroscopy (HPLC-ESI/MS) was also used. Rutin (**6**), isoquercitrin (**3**), astragalin (**20**), and isorhamnetin (**8**) were determined as major anti-oxidant compounds [77].

The in vitro, anti-oxidant activity of an alkaloid extract of *S. oppositofolia*, *S. soda* and *S. tragus* was determined by the DPPH method, using ascorbic acid (IC_50_ 2 µg/mL) as a positive control. The results revealed a significant anti-oxidant effect, with an IC_50_ value of 16.3 µg/mL, for *S. oppositifolia*. In comparison, *S. soda* and *S. tragus* extracts exhibited an IC_50_ value of 24.3 µg/mL and 26.2 µg/mL, respectively [112].

The significant anti-oxidant activity of the aqueous ethanolic extract of *S. cyclophylla* aerial parts is expressed as DPPH free-radical scavenging reactivity at IC_50_ 0.615 ± 0.06 mg/mL) [36].

*S. soda* afforded rutin (**6**), quercetin-3-*O*-glucuronopyranoside (**3**), isorhamnetin-3-*O*-rutinoside (**13**), and isorhamnetin-3-*O*-glucuronopyranoside (**10**) as major constituents. These compounds proved to be helpful in the management of diabetic problems, inflammatory diseases, and medication resistance to anthracycline-based anti-cancer therapy [81].

### 5.6. Contraceptive Effect

It is usually possible to classify contraceptive methods as either traditional or modern. Herbal medicine has always supported the potential health benefits of plants. Today, they are highly regarded as a source of safe phyto-pharmaceuticals [67].

Oral administration of the ethanolic extract (cold maceration in 70% ethanol) of the whole plant of *S. imbricata* at two doses (250 and 500 mg/kg b.wt) over a 65-day period was used to examine the contraceptive effect in male albino rats. Prior to biological evaluation, an acute toxicity study was conducted to ensure its safety. It was found to be safe up to a dose of 5 g/kg. The male contraceptive activity was related to its phenolic contents, especially quercitrin (**5**) [67].

### 5.7. Anti-Spasmodic and Bronchodilator Activity

Constipation and indigestion are two of the most frequent ailments. Constipation affects up to 27% of the population, while indigestion affects 11–29.2% of the population [85,113]. There is growing evidence that several compounds present in medicinal plants have the ability to treat gastrointestinal diseases such as indigestion and constipation in a synergistic manner [114,115]. Furthermore, medicinal plants are thought to be generally safe and beneficial when used for a long time, particularly in individuals with chronic gut motility issues.

Ethyl acetate extract of *S. collina* has significant prokinetic activity. It was effective in vivo, in promoting gastric-emptying and small-intestinal propulsion in normal male Sprague Dawley rats, showing a dose-dependent effect via a mechanism that mainly involves modulating plasma ghrelin and gastrin, as well as the expression of vasoactive intestinal peptide receptor 2 in the duodenum. In vitro, atropine promoted the contraction of both normal and relaxed gastric antrum strips, thus activating M-cholinergic receptor. This establishes a pharmacological foundation for treating gastrointestinal motility problems with *S. collina* extract [99].

Total extract, as well as the EtOAc and aqueous fractions of *S. imbricata,* caused relaxation effect on gut and tracheal tissues through the Ca^2+^ antagonist, as well as *β*-adrenergic receptor agonist effects. This explains its medicinal value in gastrointestinal and respiratory problems such as stomach colic, diarrhea, cough, and asthma [116]. The ethyl acetate fraction was found to be more effective in relaxing smooth muscle spasms than the original extract and its aqueous fraction.

The 80% ethanol extract of the whole plant of *S. baryosma* growing in Cholistan desert demonstrated anti-spasmodic activity in isolated rabbit jejunum preparations. When compared to the control verapamil, it also suppressed K+-induced contractions by 70% at 1–5 mg/mL, implying a calcium-channel-blocking activity [48].

### 5.8. Anti-Ulcer Activity

GIT disorders, which are among the leading causes of human illness, are widespread public health issues worldwide [117]. *S. imbricata* has a legendary reputation for treating a variety of gastrointestinal problems [116].

The alcoholic extract (70% alcohol in H_2_O) of the aerial parts of *S. tetrandra* showed an ulcer-protective effect like that of Ranitidine against Aspirin-induced gastric ulceration in rats in a dose-dependent manner. The ulcer index significantly decreased (*p* < 0.05) in the *Salsola*-treated rats, according to histopathological and histochemical data. In contrast, stomach mucus production increased while mucosa erosion decreased [82].

The ameliorating effect of 500 mg/kg of 50% alcohol extract of *S. komarovii* against gastritis and gastric ulcers induced by the HCl-ethanol-gastritis model was studied. It showed inhibitory effects against gastritis and gastric ulcers, which were more potent than 300 mg/kg of Ranitidine and could be used to develop a novel anti-gastric ulcer medication [37].

### 5.9. Anthelmintic Activity

The isoflavonoids 5,3′-dihydroxy-7,8,2′-trimethoxyisoflavone (**32**), 5,3′-dihydroxy-2′-methoxy-6,7-methylenedioxyisoflavone (**33**), and 5,3′-dihydroxy-6,7,8,2′-tetramethoxyisoflavone (**34**) were isolated from the *S. somalensis* roots and showed a modest anthelmintic effect in earthworms [24,27].

### 5.10. Cytotoxic Activity

The ethanol extract of *S. collina* was shown to have anti-cancer properties on human colon carcinoma HT29 cells in a dose-dependent manner by cell-cycle regulation [109]. Different fractions (*n*-hexane, CH_2_Cl_2_, EtOAc and diethyl ether) and isolated flavonols (from EtOAc fraction) from *S. oppositifolia* aerial parts were evaluated for their cytotoxic activity against five human tumor cell lines: renal adenocarcinoma ACHN, hormone-dependent prostate carcinoma LNCaP, human breast adenocarcinoma MCF-7, amelanotic melanoma C32 and large cell lung carcinoma COR-L23. The *n*-Hexane fraction was more selective against lung carcinoma compared with amelanotic melanoma cell lines, with IC_50_ values of 19.1 μg/mL and 24.4 μg/mL, respectively. Lower activity was found against renal adenocarcinoma and hormone-dependent prostate carcinoma cells (IC_50_ value of 43.4 μg/mL and 45.1 μg/mL, respectively). Additionally, the dichloromethane fraction showed the most interesting biological activity on large-cell lung carcinoma (IC_50_ 30.4 μg/mL) and amelanotic melanoma cells (IC_50_ 33.2 μg/mL). Against renal adenocarcinoma and hormone-dependent prostate cancer cells, comparable results to the *n*-hexane fraction were found (IC_50_ values of 40.4 μg/mL and 41.9 μg/mL, respectively). Meanwhile, the EtOAc fraction exhibited a cytotoxic activity, with IC_50_ values ranging from 56.4 μg/mL against amelanotic melanoma to 88.6 μg/mL against renal adenocarcinoma cells. Interestingly, a selective cytotoxic activity was demonstrated against human breast adenocarcinoma cells (IC_50_ 67.9 μg/mL) compared to other fractions. The major active constituents of this fraction were isorhamnetin-3-*O*-glucoside (**9**) and isorhamnetin-3-*O*-rutinoside (**13**), which showed an interesting activity against human breast adenocarcinoma cell line, with IC_50_ values of 18.2 and 25.2 μg/mL, respectively. Moreover, isorhamnetin-3-*O*-glucoside (**9**) showed good cytotoxic activity against the renal adenocarcinoma and the hormone-dependent prostate carcinoma cells, with IC_50_ values of 26.1 and 28.5 μg/mL, respectively. Isorhamnetin-3-*O*-rutinoside (**13**) exhibited potent activity against the hormone-dependent prostate carcinoma cell line, with an IC_50_ of 20.5 μg/mL. Diethyl ether fraction was selective against the renal adenocarcinoma cell line (IC_50_ values of 46.8 μg/mL). The remarkable cytotoxic effect of the two non-polar fractions (*n*-hexane and diethyl ether), specifically against COR-L23 and C32 cells, may be attributed to the presence of fatty acids and methyl esters, based on their chemical makeup [80].

The IC_50_ of the ethyl acetate fraction of the whole plant of *S. baryosma* was determined using a brine shrimp assay, and the number of larvae that survived after the addition of various amounts of test sample, using Permethrin (236 g/cm^3^) as a standard, was calculated to be 1 mg/mL. On the other hand, all fractions of *S. baryosma* (ethanol 80%, *n*-hexane, EtOAc and *n*-BuOH) were found to be phytotoxic to a varying degree, from 52% to 100%, which was assessed by the inhibition of *Lemna minor* plant growth in a dose-dependent manner, using paraquat as standard drug (0.9025 µg/mL) [48]. Finally, taxiphyllin (**229**) from *S. tetrandra* showed high cytotoxic activity in the *Artemia salina* lethality bioassay, with an ED_50_ value of 0.96 µM [70]. Likewise, most cytotoxic results are based on cell-based inhibition, with no tumor xenografted animal model to prove efficacy. This should be considered as a next step.

### 5.11. Vaso-Activity Effect

The ethanol extract of *Salsola* was shown to have hypotensive activity in rats, induced by Nω-Nitro-L-Ariginine (L-NNA)[118].

The alkaloids salsoline (**110**) and salsolidine (**112**) were isolated from *S. kali* and used for the treatment of hypertonia, hypertension and headache (as hydrochloride) by stimulating the activity of sleep and as a nervous system tonifier [12].

Captopril was used as a reference ACE inhibitor to examine the ethyl acetate extracts of the aerial parts of *S. oppositifolia*, *S. soda*, and *S. tragus* for their hypotensive activities. With IC_50_ values of 181.04 and 284.27 g/mL, *S. oppositifolia* and *S. soda* showed an interesting suppression of ACE activity. *S. tragus*, on the other hand, showed minimal action, with an inhibition percentage of 36.21 ± 0.4%. Furthermore, using water as a negative control, a gelatin salt block test was used to reduce the false-positive effect caused by tannins. Thus, tannins are not the only factor affecting the efficacy of *S. oppositifolia* and *S. soda* EtOAc extracts in inhibiting ACE [74].

### 5.12. Hypoglycemic Effect

The hypoglycaemic effects of methanol extract of the aerial parts of *S. kali*, *S. soda*, and *S. oppositifolia* were evaluated in vitro using an in vitro assay based on the suppression of the *α*-amylase digesting enzyme. The ethyl acetate fraction of the extract was the most active, with an IC_50_ value of 0.022 mg/mL.

In addition, *N*-acetyltryptophan (**121**), which is a derivative of amino acid and was isolated from *S. collina,* showed a moderate inhibition of *α*-amylase activity using the Caraway iodine/potassium iodide (IKI) method [47].

### 5.13. Anti-Acetylcholinesterase and Anti-Butyrylcholinesterase Activity

Triterpene salsolic acid (**140**) was isolated from the chloroform fraction of *S. baryosma,* and showed inhibitory activity against the enzyme butyrylcholinesterase (BChE) [43,44]. Moreover, amino acid derivative, *N-*acetyltryptophan (**121**), which was isolated from *S. grandis*, displayed a marked inhibitory activity against acetylcholinesterase (AChE) (64.90 ± 1.61%) at a dose of 50 µg/mL using a microtiter assay. Moreover, molecular modelling experiments were performed. The interactions between *N*-acetyltryptophan (**121**), at the atomic level, and AChE, were established using in silico experiments. Thus, *N-*acetyltryptophan (**121**) could be a valuable preclinical molecule for AChE inhibitors, with neuroprotective potential, especially in the treatment of Alzheimer’s disease (AD) [50].

Moreover, due to high catecholamine content in their *S. vermiculata’*s roots, they could also inhibit AChE- with an IC_50_ value of 0.45 ± 0.17 mg/mL, which is comparable with that of Eserine (physostigmine) [26].

Moreover, alkaloid fractions prepared from *S. oppositofolia*, *S. soda*, and *S. tragus* aerial parts showed promising activity against acetylcholinesterase (AChE) and BChE enzymes. The *S. tragus* activity was the highest against AChE and BchE (with an IC_50_ of 30.2 g/mL and IC_50_ of 26.5 g/mL, respectively). Meanwhile, with IC_50_ values of 34.3 g/mL and 32.7 g/mL, respectively, *S. soda* and *S. oppositifolia* alkaloid fractions had a specific inhibitory action against BChE. The high activity of *S. tragus* against AChE and BChE enzymes could be due to its high alkaloids salsoline (**110**) (36.5%) and salsolidine (**112**) (17.7%) contents [112].

Other components in the *Salsola* matrix with higher specific activity may, however, perform additively or synergistically, and may eventually be relevant in the anti-acetylcholinesterase effect [26].

### 5.14. Neuroprotective Activity

Exogenous nerve growth factor (NGF) improves the cholinergic neuron system and has therapeutic potential for neurodegenerative disorders such as Parkinson’s disease, Alzheimer’s disease, and diabetic polyneuropathy. Nineteen compounds isolated from the MeOH extract of the aerial parts of *S. komarovii* were tested on C6 glial cells to see how they affected NGF induction. Cell viability was determined by MTT assay, and 6-Shogoal was used as a positive control. (*8S,8′R,7′R*)-9′-[(*β*-glucopyranosyl)oxy] lyoniresinol (**197**) was a stimulant for NGF secretion in C6 cells (127.3 ± 10.3%) but was cytotoxic at low concertations. Additionally, alangilignoside C (**199**), conicaoside (**200**) and blumenyl B *β*-D-glucopyranoside (**206**) were found to upregulate (NGF) secretion without significant cell toxicity. The most effective stimulator of NGF release, conicaoside (**200**), may have neuroprotective properties by stimulating NGF secretion [41].

### 5.15. Tyrosinase Inhibitory Activity

The three isolated phenolic compounds, *N*-[2′-(3″,4″-dihydroxyphenyl)-2′-hydroxyethyl]-3-(4‴-methoxyphenyl)prop-2-enamide (**99**), *N*-[2′-(3″,4″-dihydroxyphenyl)-2′-hydroxyethyl]-3-(3‴,4‴-dimethoxyphenyl)prop-2-enamide (**100**) and *N*-[2′-(3″-hydroxy-4″-methoxyphenyl)-2′-hydroxyethyl]3-(4‴-methoxyphenyl)-prop-2-enamide (**101**) from the whole plant of *S. baryosma*, were studied for their ability to inhibit mushroom tyrosinase. They exhibited pronounced tyrosinase inhibition activity, with an IC_50_ of 2.61, 1.85, and 0.40 µM, respectively. As a result, *S. baryosma* can be utilized to treat disorders such as hyperpigmentation, caused by excessive melanocyte production [55].

### 5.16. Other Activities

Many species of this genus can act as an allergenic substance [119]. *S. baryosma* is used as a diuretic agent, vermifugal, and the ash is applied to itches [87]. Furthermore, an aqueous extract of *S. collina* is an effective means of cholelithiasis prophylaxis by: (i) inhibiting the development of inflammation in the mucous membrane of the gallbladder against the background of an aggressive atherogenic diet; (ii) favoring cholesterol absorption by the mucous membrane of the gallbladder; (iii) stimulating the absorption of water, thus maintaining a high concentration of bile acid in the gallbladder bile; (iv) preventing the precipitation of calcium allodeoxycholate crystals and the formation of a biliary slough [120].

### 5.17. As a Fodder

*Salsola* species, especially in the autumn and winter in deserts, can be utilized as a partial substitute for feed concentrates. The aerial parts of *S. cyclophylla*, which grow in marshy areas of central Saudi Arabia, are frequently used for both medicinal and feeding purposes [39], as a potential alternative food supply during food shortages and drought times [36], and as nutraceuticals. This was corroborated by the richness of phytoconstituents such as flavonoids and phenols [39]. Moreover, *Salsola* species are a promising camel feed in Pakistan’s Cholistan desert [121]. Their development as a viable fodder species in arid regions was aided by a number of characteristics such as excellent nutritional qualities, prolific seed production, resistance to high temperatures, and long-term drought tolerance [122].

## 6. Conclusions and Future Prospective

A major driving force for drug discovery over the last century has involved utilizing natural products and their metabolites as a chemically diverse starting building block. The application of natural products, however, is not limited to the modern era, as most traditionally used crude drugs (remedies) have plant-derived extracts. Furthermore, the advancement of modern technologies and the ability to isolate and identify the natural bioactive ingredient in plants, have encouraged researchers to explore and apply them in food and nutraceuticals, as well as medicine.

The genus *Salsola*, known to be widespread worldwide, has a history of medicinal uses against different diseases in the folk medicine system of several civilizations. In this review, the authors rediscover the genus *Salsola* by highlighting the important isolated and identified chemical compounds and extracts, along with their reported biological activities. For example, salsolic acid (**140**), which was isolated from *S. baryosma*, showed inhibitory activity against (BChE). Meanwhile, *N*-acetyl tryptophan (**121**), which was isolated from *S. grandis*, displayed a marked inhibitory activity against (AchE). Thus, it might be a promising precursor model with neuroprotective potential. In addition, compounds (**197**, **199**, **200**, **206**), isolated from the methanol extract of the aerial parts of *S. komarovii*, were found to be a potent stimulant of (NGF) secretion, with potential neuroprotective activity and without significant cell toxicity. Thus, this has therapeutic potential for neurodegenerative diseases, and particularly for (AD) treatment. The three phenolic compounds (**99**–**101**) isolated from the whole plant of *S. foetida* exhibited pronounced tyrosinase inhibition activity, with the potential to be used for the treatment of diseases such as hyper-pigmentation, associated with the overproduction of melanocytes. These bioactive molecules could be used as a starting material in drug discovery for treatment of the aforementioned diseases.

Promising activity was also observed for some *Salsola* species. The alkaloid fraction of *S. tragus* showed promising activity against both AChE and BChE enzymes and could be a source of drug lead in AD treatment. Different fractions (*n*-hexane, CH_2_Cl_2_, EtOAc and diethyl ether) and isolated flavonols from the EtOAc fraction of *S. oppositifolia* aerial parts exhibited promising in vitro cytotoxic activity against five human tumor cell lines: ACHN, LNCaP, MCF-7, COR-L23 and C32. Moreover, the ethanol extract of *S. collina* showed anti-cancer activity on human colon carcinoma HT29 cells in a dose-dependent manner by cell regulation. Ulcer-protective effects such as Ranitidine’s effect against aspirin-induced gastric ulceration were found in the alcoholic extract of the aerial parts of *S. tetrandra*. Moreover, the EtOAc fraction of aerial parts of *S. oppositifolia* and *S. soda*, together with compounds (**110**, **112**), found in *S. kali*, showed hypotensive activity.

Whilst most studies of the bioassays of *Salsola* extracts or its isolated compounds focused on in vitro cell-based assays, few have attempted to use animal models to confirm efficacy. These studies should now follow, so that the results are conclusive. Likewise, profiling halophytes of a different geographical origin can reveal how different environments can affect *Salsola*’s chemistry and or biological effects. The application of metabolomic approaches for the large-scale profiling of the genus, to provide a holistic assessment of its metabolite chemical composition, has been little reported in the literature compared to other medicinal plants. The optimization of extraction methods that would aid in recovering the highest yield of its bioactive compounds should be attempted, considering its high salt levels, which could hinder the detection and or identification of active agents. Indeed, identification of the best extraction strategies for halophytes is much more limited than that reported for other plant phyla.

Additionally, plants in the genus *Salsola* have long been used in traditional medicine to treat a variety of ailments that have yet to be pharmacologically proven. Standardization of these traditionally used plants will facilitate their incorporation in nutraceuticals. Most of the published research has concentrated on the chemistry and pharmacology of the aerial parts, with only a few publications on the roots encouraging researchers to investigate them further. Since cinnamate esters have been found in a variety of *Salsola* species, the presence of benzoate esters in *S. cyclophylla* suggests the need for further studies on the biosynthetic pathways involved in the production of benzoates versus cinnamates. While rosmarinic acid (**87**) is common in the Lamiaceae family, its presence in *S. imbricata* necessitates greater research into biosynthesis pathways, which can help further agronomic and molecular approaches to improve its yield. Moreover, a detailed phytochemical profiling, in parallel with gene expression, could help to establish different biosynthetic pathways in different organs. While isoflavones are restricted to a few plant families, mostly legumes, they have been detected in the roots of *S. somalensis*, *S. tetrandra* and leaves of *S. imbricata*; their ecological role in *Salsola* has yet to be determined. Whether cardinolides only exist in *S. tetragona*, or if they occur in other *Salsola* species, needs to be confirmed by profiling many other species for comparison. Finally, few studies have been presented on the volatile composition in *Salsola* species; this should be compared to that reported in *S. cyclophylla* and *S. vermiculate* in the future.

## Figures and Tables

**Figure 4:**
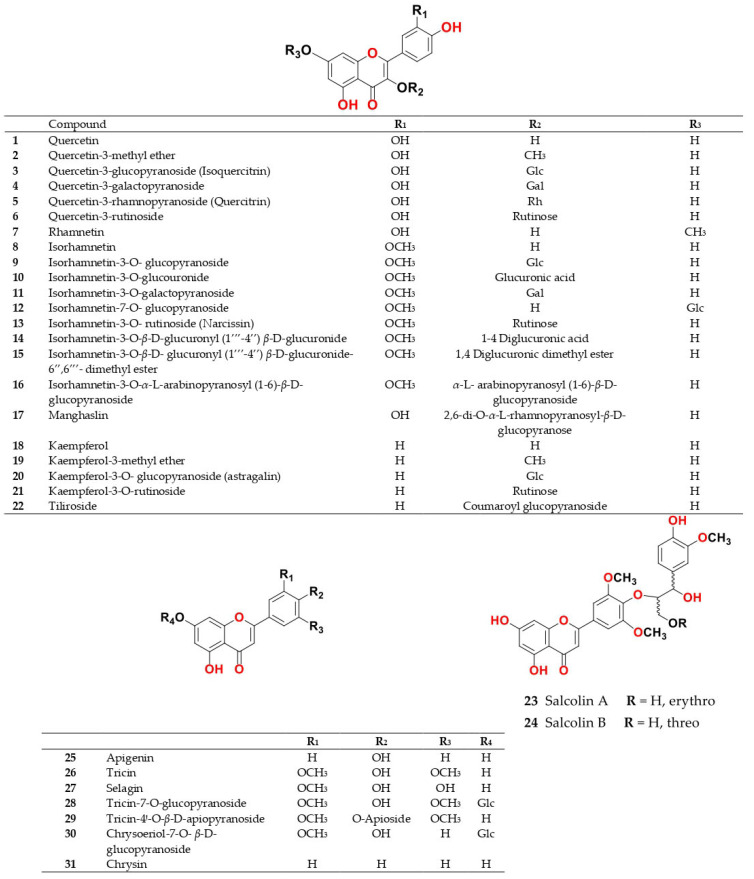
Chemical structure of flavonoids (**1**–**31**) isolated from genus *Salsola*.

**Figure 5:**
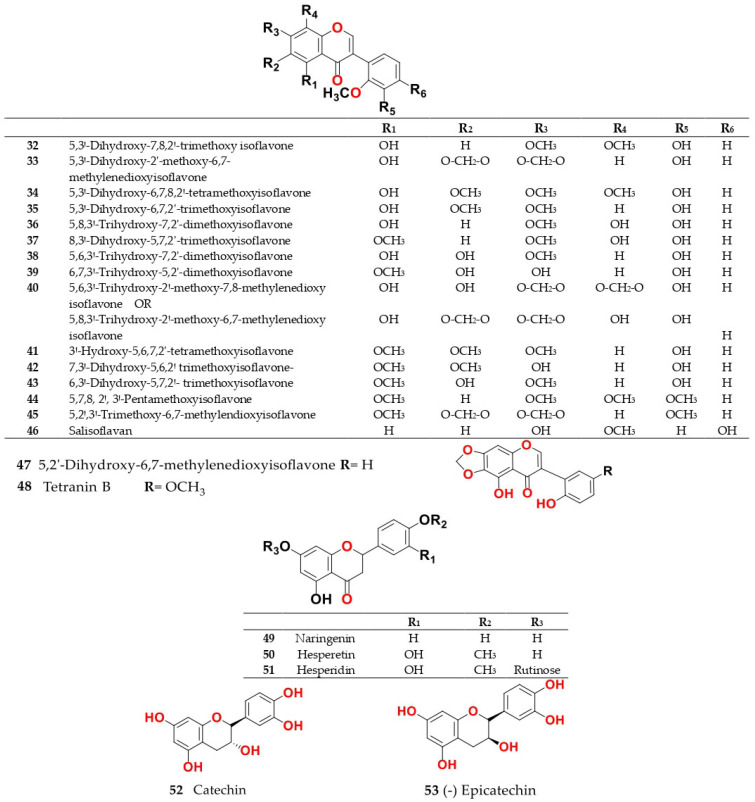
Chemical structure of flavonoids (**32**–**53**) isolated from genus *Salsola*.

**Figure 6:**
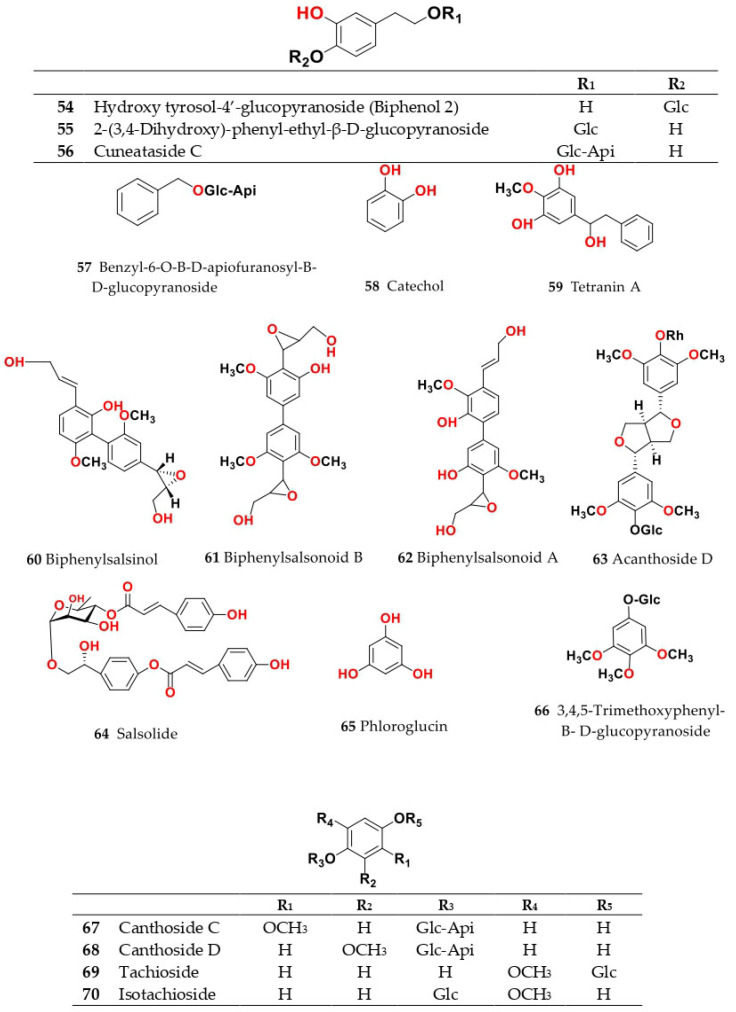
Chemical structure of phenolic compounds isolated from genus *Salsola.*

**Figure 7:**
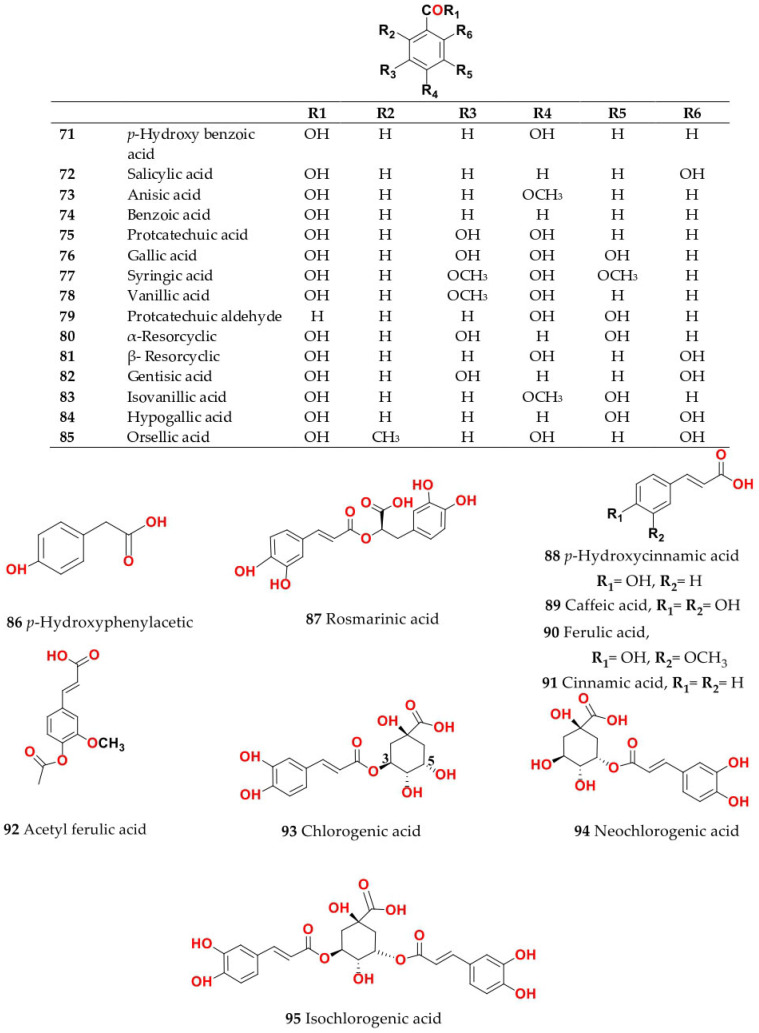
Chemical structure phenolic acids isolated from genus *Salsola.*

**Figure 8:**
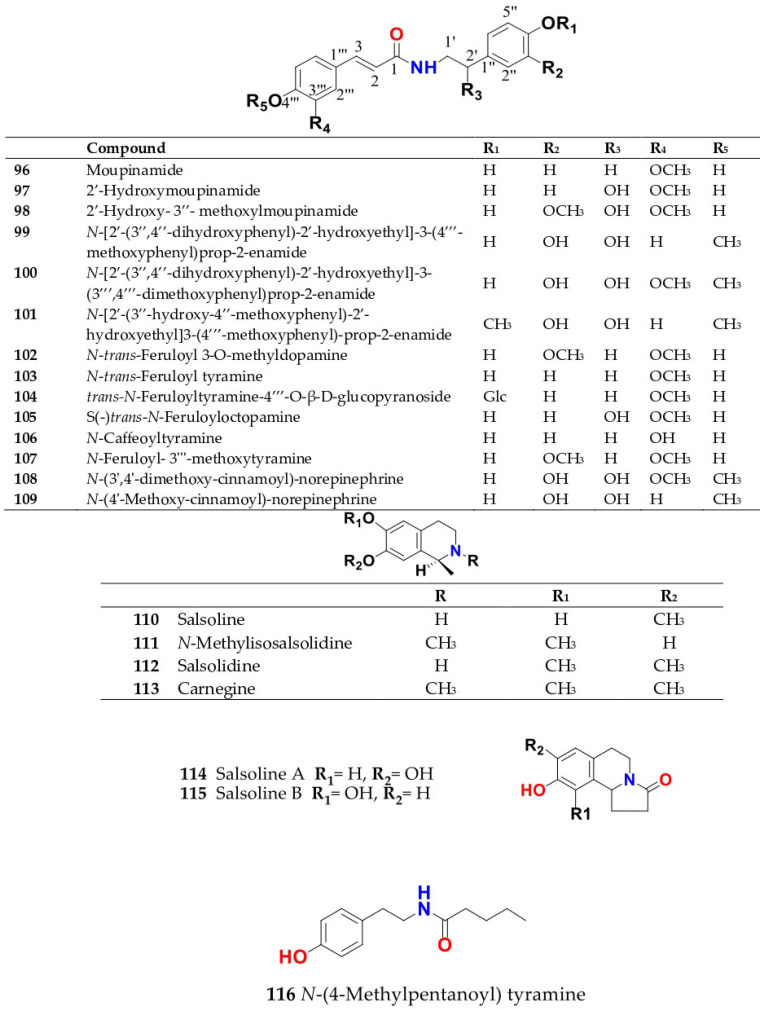
Chemical structure of nitrogenous compounds (**96**–**116**) isolated from genus *Salsola*.

**Figure 9:**
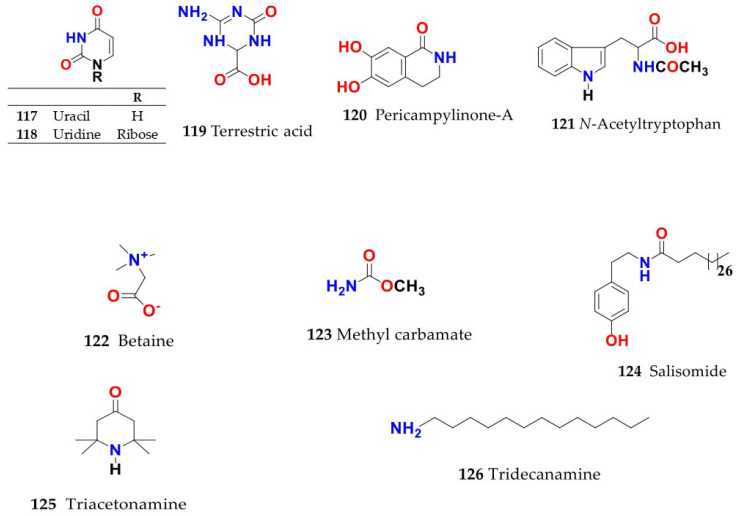
Chemical structure of nitrogenous compounds (**117**–**126**) isolated from genus *Salsola.*

**Figure 10:**
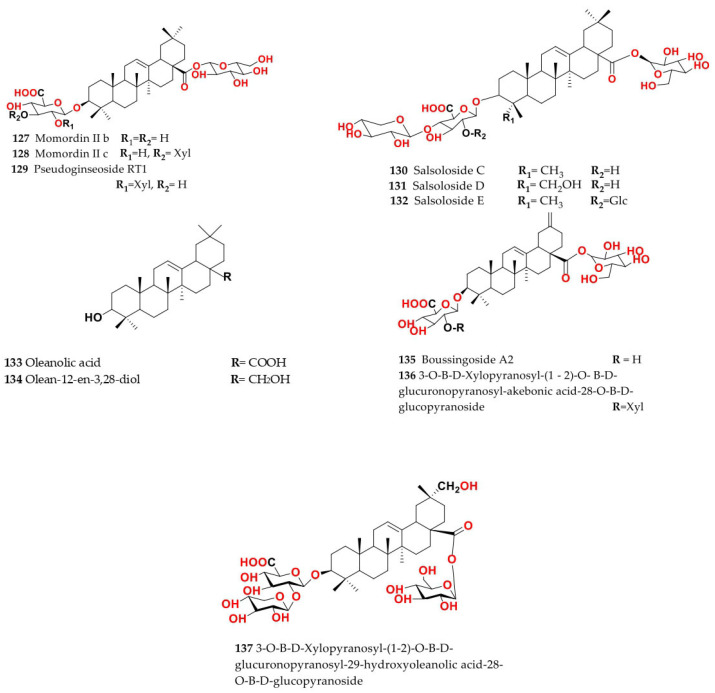
Chemical structure of saponins isolated from genus *Salsola.*

**Figure 11:**
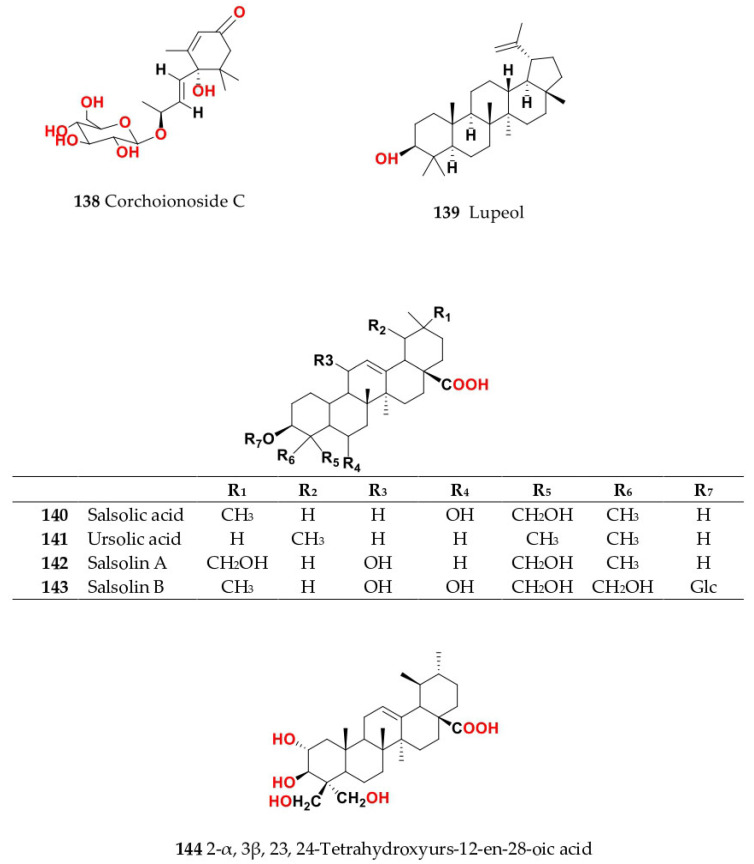
Chemical structure of terpenoid compounds isolated from genus *Salsola.*

**Figure 12:**
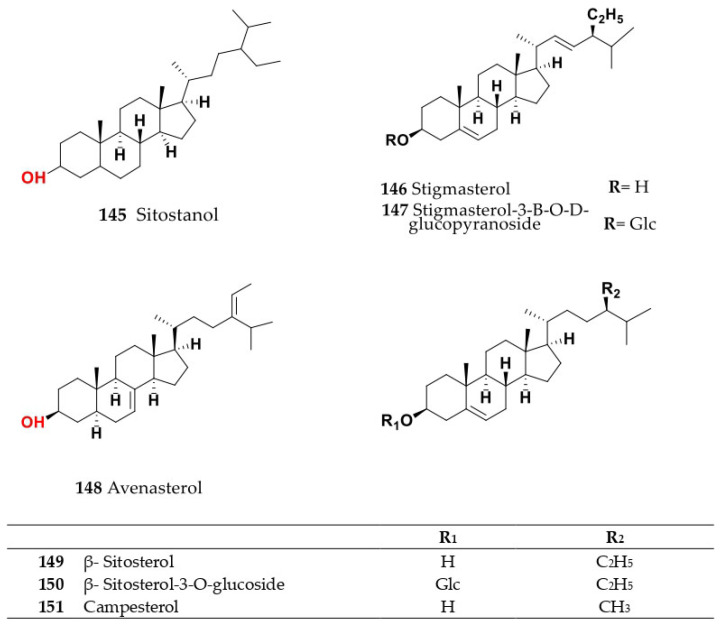
Chemical structure of sterols isolated from genus *Salsola.*

**Figure 13:**
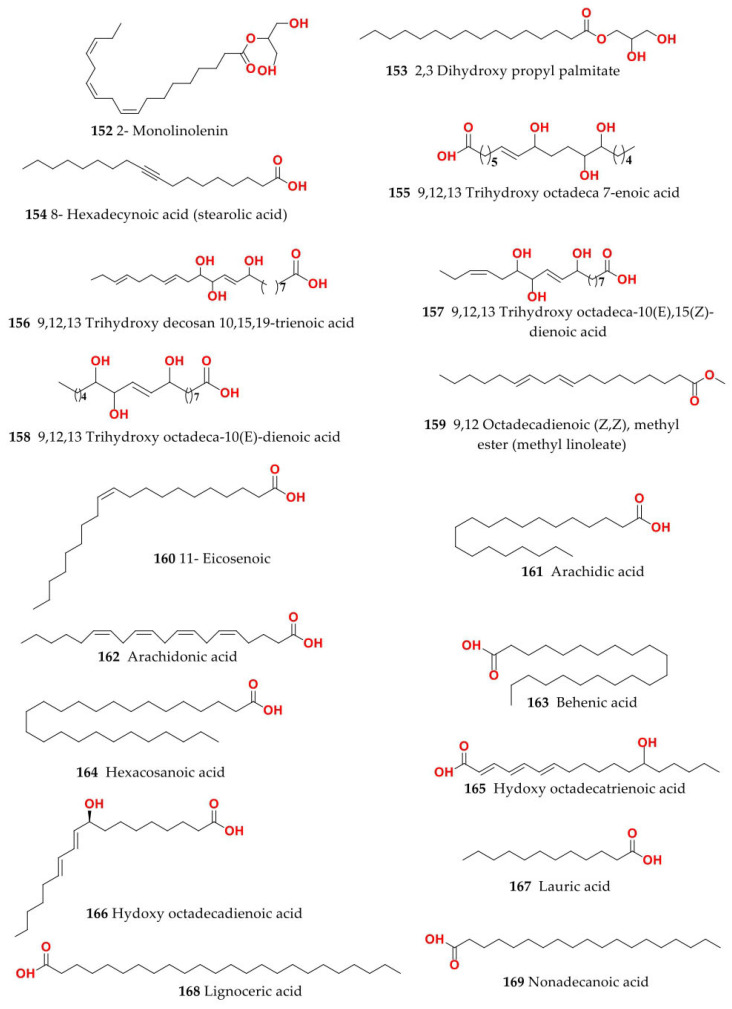
Chemical structure of fatty acids (**152**–**169**) isolated from genus *Salsola.*

**Figure 14:**
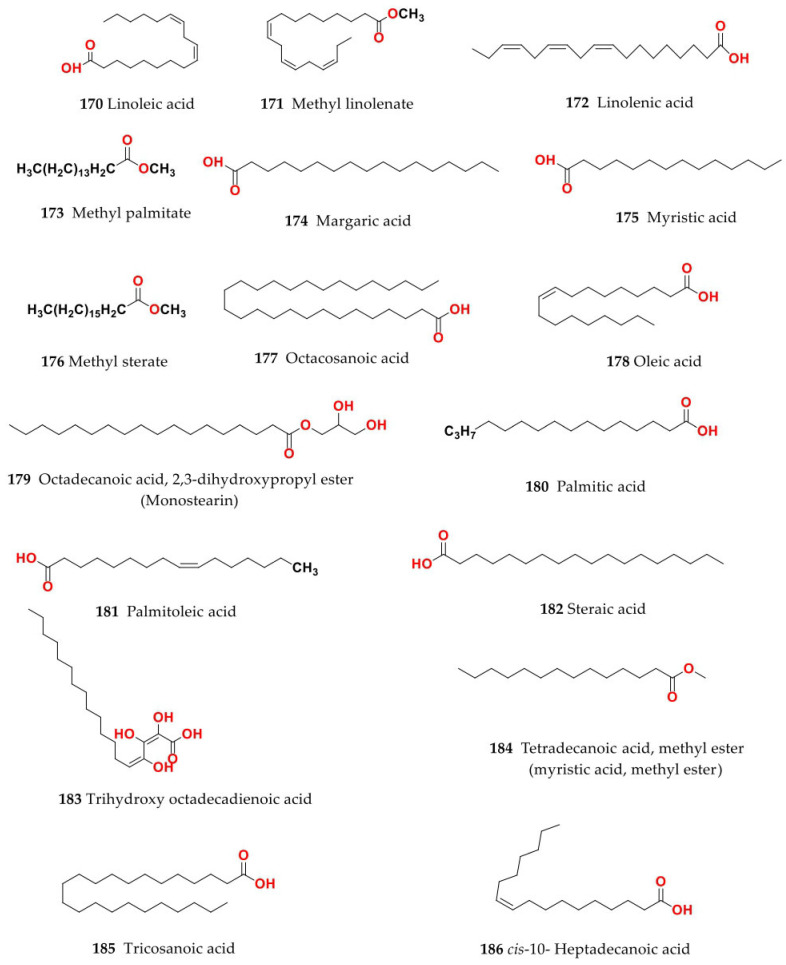
Chemical structure of fatty acids (**170**–**186**) isolated from genus *Salsola.*

**Figure 15:**
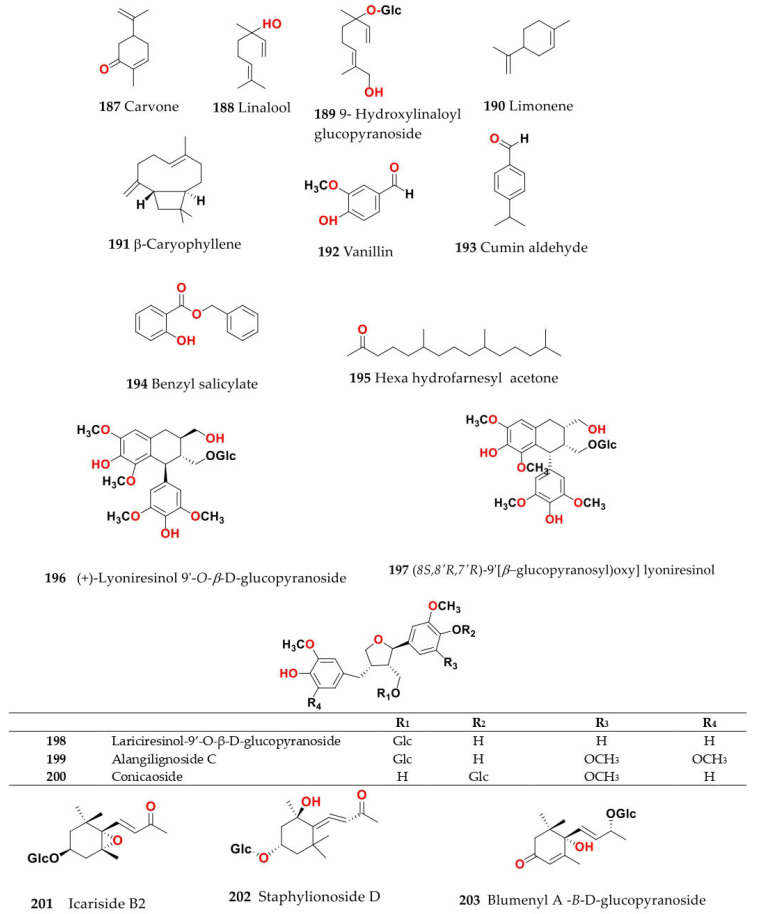
Chemical structure of volatile constituents, their glycosides, lignans and megastegmanes, isolated from genus *Salsola.*

**Figure 16:**
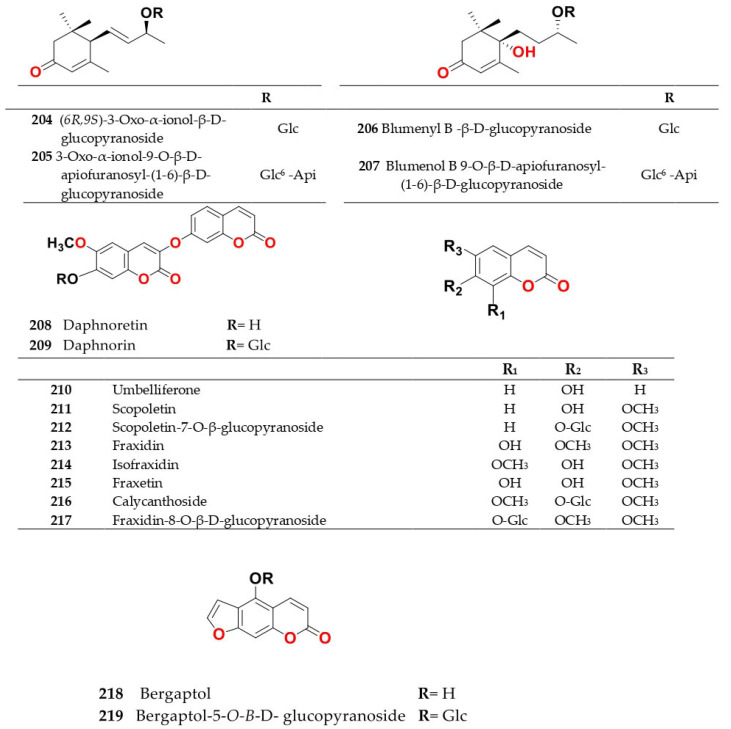
Chemical structure of megastegmanes (cont.) and coumarins isolated from genus *Salsola.*

**Figure 17:**
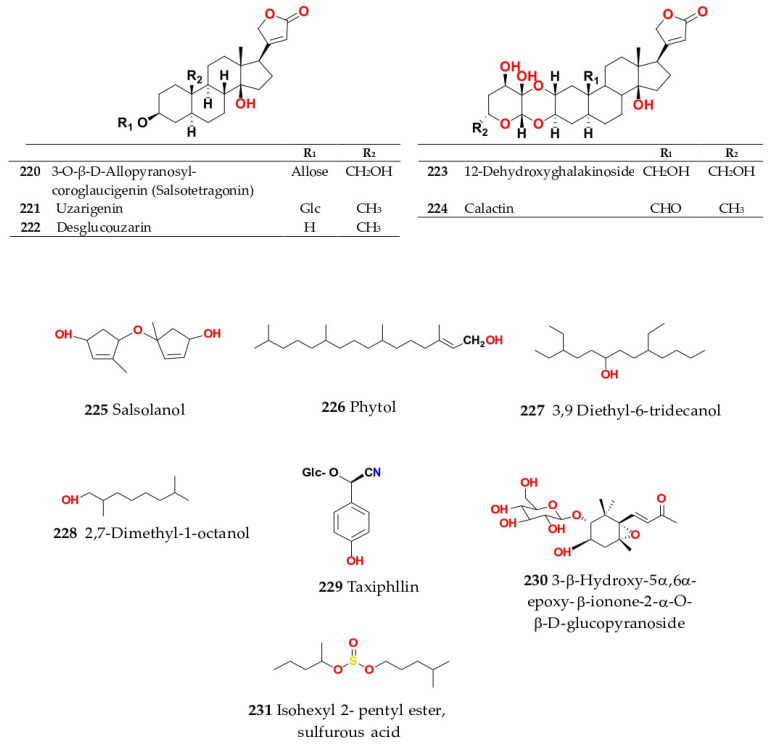
Chemical structure of cardiac glycoside-, alcohol-, cyanogenic-, isoprenoid-, and Sulphur-containing compounds isolated from genus *Salsola.*

**Table 1:** List of accepted species in genus *Salsola* and their synonyms [16,18].

Accepted Species in Genus *Salsola*	Synonyms
*Salsola acanthoclada* Botsch.	*Nitrosalsola acanthoclada* (Botsch.) Theodorova
*Salsola africana* (Brenan) Botsch.	*Salsola dendroides* var. *africana* Brenan
*Salsola algeriensis* Botsch.	*Nitrosalsola algeriensis* (Botsch.) Theodorova
*Salsola angusta* Botsch.	-
*Salsola arbusculiformis* Drobow	-
*Salsola australis* R.Br.	*Kali australe* (R.Br.) Akhani and Roalson *Kali macrophyllum* (R.Br.) Galasso and Bartolucci *Salsola macrophylla* R.Br. *Salsola tragus* var. *australis* (R.Br.) Bég.
*Salsola austroiranica* Akhani	-
*Salsola austrotibetica* Sukhor.	-
*Salsola baranovii* Iljin	-
*Salsola brevifolia* Desf.	*Nitrosalsola brevifolia* (Desf.) Theodorova
*Salsola chellalensis* Botsch.	*Nitrosalsola chellalensis* (Botsch.) Theodorova
*Salsola chinghaiensis *A.J.Li	-
*Salsola collina* Pall.	*Kali collinum* (Pall.) Akhani and Roalson *Salsola chinensis* Gand. *Salsola erubescens* Schrad. *Salsola ircutiana* Gand. *Salsola kali* subsp. *collina* (Pall.) O.Bolòs and Vigo
*Salsola cruciata* L.Chevall. ex Batt. and Trab.	*Darniella cruciata* (L.Chevall. ex Batt. and Trab.) Brullo
*Salsola cyrenaica* (Maire and Weiller) Brullo	*Darniella cyrenaica* Maire and Weiller *Salsola sieberi* subsp. *cyrenaica* (Maire and Weiller) Brullo and Furnari
*Salsola daghestanica* (Turcz. ex Bunge) Lipsky	*Noaea daghestanica* Turcz. ex Bunge
*Salsola divaricata* Masson ex Link	*Salsola capensis* Botsch. *Salsola divaricata* (Moq.) Ulbr.
*Salsola drummondii* Ulbr.	*Salsola obpyrifolia* Botsch. and Akhani
*Salsola euryphylla* Botsch.	-
*Salsola foliosa* (L.) Schrad. ex Schult.	*Anabasis clavata* S.G.Gmel. *Anabasis foliata* Pall. ex Bunge *Anabasis foliosa* L. *Caspia foliosa* (L.) Galushko *Micropeplis foliosa* (L.) G.L.Chu *Neocaspia foliosa* (L.) Tzvelev *Salsola baccifera* Pall. *Salsola clavifolia* Pall.
*Salsola glomerata* (Maire) Brullo	*Darniella glomerata* (Maire) Brullo
*Salsola gobicola* Iljin	*Kali gobicola* (Iljin) Brullo and Hrusa
*Salsola grandis* Freitag, Vural and Adigüzel	-
*Salsola griffithii* (Bunge) Freitag and Khani	*Kali griffithii* (Bunge) Akhani and Roalson *Noaea griffithii* Bunge
*Salsola gymnomaschala* Maire	*Darniella gymnomaschala* (Maire) Brullo *Seidlitzia gymnomaschala* (Maire) Iljin
*Salsola gypsacea* Botsch.	
*Salsola halimocnemis* Botsch.	*Nitrosalsola gypsacea* (Botsch.) Theodorova
*Salsola hartmannii* Sukhor.	-
*Salsola ikonnikovii* Iljin	*Kali ikonnikovii* (Iljin) Akhani and Roalson *Salsola beticolor* Iljin *Salsola centralasiatica* Iljin *Salsola potaninii* Iljin
*Salsola jacquemontii* Moq.	*Kali jacquemontii* (Moq.) Akhani and Roalson *Kali nepalensis* (Grubov) Brullo, Giusso and Hrusa *Salsola nepalensis* Grubov
*Salsola junatovii* Botsch.	-
*Salsola kali* L.	*Corispermum pilosum* Raf. *Kali soda* Moench *Kali turgidum* (Dumort.) Gutermann *Salsola acicularis* Salisb. *Salsola aptera* Iljin *Salsola decumbens* Lam. *Salsola gmelinii* Rouy *Salsola kali* var. *apula* Ten. *Salsola kali* subsp. *austroafricana* Aellen *Salsola kali* var. *hirta* Ten. *Salsola kali* var. *mixta* W.D.J.Koch *Salsola kali* var. *rosacea* Pall. *Salsola kali* var. *rosacea* Moq. *Salsola kali* var. *rubella* Moq. *Salsola kali* var. *vulgaris* W.D.J.Koch *Salsola scariosa* Stokes *Salsola spinosa* Lam. *Salsola turgida* Dumort.
*Salsola kerneri* (Wol.) Botsch.	-
*Salsola komarovii* Iljin	*Kali komarovii* (Iljin) Akhani and Roalson
*Salsola laricifolia* Litv. ex Drobow	-
*Salsola longifolia* Forssk.	*Darniella longifolia* (Forssk.) Brullo *Darniella sinaica* (Brullo) Brullo *Salsola fruticosa* Cav. *Salsola longiflora* J.F.Gmel. *Salsola oppositifolia* Sieber ex Moq. *Salsola sieberi* C.Presl *Salsola sinaica* Brullo *Seidlitzia longifolia* (Forssk.) Iljin
*Salsola mairei* Botsch.	*Nitrosalsola mairei* (Botsch.) Theodorova
*Salsola makranica* Freitag	-
*Salsola masclansii* G.Monts. and D.Gómez	-
*Salsola melitensis* Botsch.	*Darniella melitensis* (Botsch.) Brullo
*Salsola monoptera* Bunge	*Kali monopterum* (Bunge) Lomon.
*Salsola omanensis* Boulos	-
*Salsola oppositifolia* Desf.	*Petrosimonia sibirica* (Pall.) Bunge
*Salsola pachyphylla* Botsch.	-
*Salsola papillosa* (Coss.) Willk.	*Salsola angularis* Sennen
*Salsola paulsenii* Litv.	*Kali paulsenii* (Litv.) Akhani and Roalson *Kali pellucidum* (Litv.) Brullo, Giusso and Hrusa *Salsola pellucida* Litv.
*Salsola pontica* (Pall.) Iliin	*Kali ponticum* (Pall.) Sukhor. *Kali tragus* subsp. *ponticum* (Pall.) Mosyakin *Salsola kali* var. *pontica* Pall. *Salsola kali* subsp. *pontica* (Pall.) Mosyakin *Salsola pontica* var. *glabra* Tzvelev *Salsola squarrosa* subsp. *pontica* (Pall.) Mosyakin *Salsola tragus* subsp. *pontica* (Pall.) Rilke
*Salsola praecox* (Litv.) Litv.	*Kali praecox* (Litv.) Sukhor. *Salsola elegantissima* Iljin *Salsola kali* var. *praecox* Litv. *Salsola paulsenii* subsp. *praecox* (Litv.) Rilke
*Salsola praemontana* Botsch.	*Nitrosalsola praemontana* (Botsch.) Theodorova
*Salsola ryanii* Hrusa and Gaskin	*Kali ryanii* (Hrusa and Gaskin) Brullo and Hrusa
*Salsola sabrinae* Mosyakin	*Salsola tragus* subsp. *grandiflora* Rilke
*Salsola schweinfurthii* Solms	*Darniella schweinfurthii* (Solms) Brullo
*Salsola sinkiangensis* A.J.Li	*Kali sinkiangense* (A.J.Li) Brullo, Giusso and Hrusa
*Salsola squarrosa* Steven ex Moq.	*Kali dodecanesicum* C.Brullo, Brullo, Giusso and Ilardi *Salsola controversa* Tod. ex Lojac. *Salsola squarrosa* subsp. *controversa* (Tod. ex Lojac.) Mosyakin
*Salsola strobilifera* (Benth.) Mosyakin	*Salsola australis* var. *strobilifera* (Benth.) Domin *Salsola kali* var. *strobilifera* Benth.
*Salsola subglabra* Botsch.	*Nitrosalsola subglabra* (Botsch.) Theodorova
*Salsola tamamschjanae* Iljin	*Kali tamamschjanae* (Iljin) Akhani and Roalson
*Salsola tamariscina* Pall.	*Caroxylon tamariscinum* (Pall.) Moq. *Kali tamariscinum* (Pall.) Akhani and Roalson *Salsola tamariscifolia* Falk *Salsola tenuifolia* Falk
*Salsola tragus* L.	*Kali tragus* (L.) Scop. *Salsola altaica* (C.A.Mey.) Iljin *Salsola brachypteris* Moq. *Salsola caroliniana* Walter *Salsola dichracantha* Kitag. *Salsola iberica* (Sennen and Pau) Botsch. ex Czerep. *Salsola kali* var. *brachypteris* (Moq.) Benth. *Salsola kali* var. *brevimarginata* W.D.J.Koch *Salsola kali* var. *caroliniana* (Walter) Nutt. *Salsola kali* var. *glabra* Ten. *Salsola kali* subsp. *iberica* (Sennen and Pau) Rilke *Salsola kali* var. *leptophylla* Benth. *Salsola kali* subsp. *ruthenica* (Iljin) Soó *Salsola kali* var. *tenuifolia* Tausch *Salsola kali* var. *tragus* (L.) Moq. *Salsola pestifer* A.Nelson *Salsola pseudotragus* (Beck) Iljin *Salsola ruthenica* Iljin *Salsola ruthenica* var. *filifolia* A.J.Li *Salsola ruthenica* var. *tragus* (L.) Morariu *Salsola tragus* subsp. *iberica* Sennen and Pau *Salsola tragus* var. *pseudocollina* Tzvelev *Salsola tragus* var. *tenuifolia* (Tausch) Tzvelev
*Salsola tunetana* Brullo	*Darniella tunetana* (Brullo) Brullo
*Salsola turcica* Yild.	-
*Salsola verticillata* Schousb.	*Darniella verticillata* (Schousb.) Brullo *Salsola deschaseauxiana* Litard. and Maire *Seidlitzia verticillata* (Schousb.) Iljin
*Salsola webbii* Moq.	*Anabasis tamariscifolia* Webb *Salsola ericoides* Lag. ex Willk. and Lange
*Salsola zaidamica* Iljin	*Kali zaidamicum* (Iljin) Akhani and Roalson
*Salsola zygophylla* Batt.	*Darniella zygophylla* (Batt.) Brullo

## Data Availability

Data available in a publicly accessible repository that does not issue DOIs “Publicly available datasets were analyzed in this study. This data can be found here: [https://powo.science.kew.org/taxon/urn:lsid:ipni.org:names:30012872-2], reference number: [18].

## Supplementary Materials

The following supporting information can be downloaded at: https://www.mdpi.com/article/10.3390/plants11060714/s1, Table S1: List of flavonoids isolated from different *Salsola* species, Table S2: List of phenolic compounds isolated from different *Salsola* species, Table S3: List of phenolic acids isolated from different *Salsola* species, Table S4: List of nitrogenous compounds isolated from different *Salsola* species, Table S5: List of saponin compounds isolated from different *Salsola* species, Table S6: List of triterpenes isolated from different *Salsola* species, Table S7: List of sterols isolated from different *Salsola* species, Table S8: List of fatty acids isolated from different *Salsola* species, Table S9: List of volatile constituents isolated from different *Salsola* species, Table S10: List of lignanas isolated from different *Salsola* species, Table S11: List of magastigmane isolated from different *Salsola* species, Table S12: List of coumarins isolated from different *Salsola* species, Table S13: List of cardiac glycosides isolated from different *Salsola* species, Table S14: List of alcohols isolated from different *Salsola* species, Table S15: List of cyanogenic, isoprenoid, sulphur-containing, and ester compounds isolated from different *Salsola* species.
