# Supplementary material for: Genus Salsola: Chemistry, Biological Activities and Future Prospective—A Review
Source: Plants (Basel). 2022 Mar 8;11(6):714. doi: 10.3390/plants11060714 (PMC8953912; doi:10.3390/plants11060714)
Supplement: Supplementary file 1 [file plants-11-00714-s001.zip › plants-1581338-supplementary.pdf]

Table S1: List of flavonoids isolated from different *Salsola* species

No	Compound Name	Source	Molecular Formula	Molecular weight g/mol	References
**1**	Quercetin	*S. kali*<br>*S. imbricata*<br>*S. baryosma (S. foetida)*<br>*S. volkensii,*<br>*S. collina,*<br>*S. grandis*<br>*S. longifolia*<br>*S. tetrandra*<br>*S. vermiculata*	$C_{15}H_{10}O_7$	302.2	[1–11]
**2**	Quercetin-3-O-methyl ether	*S. grandis*	$C_{16}H_{12}O_7$	316.3	[3,4]
**3**	Quercetin-3-O-glucoside (Isoquercitrin)	*S. kali*<br>*S. volkensii*<br>*S. tetragona*<br>*S. collina*<br>*S. soda*<br>*S. komarovii*	$C_{21}H_{20}O_{12}$	464.4	[8,12–15]
**4**	Quercetin-3-O-glactoside (Hyperoside)	*S. grandis*	$C_{21}H_{20}O_{12}$	464.4	[3,4]
**5**	Quercetin-3-O-rhamnoside (Quercitrin)	*S. kali*<br>*S. longifolia*<br>*S. grandis*<br>*S. imbricata*	$C_{21}H_{20}O_{11}$	448.4	[3,4,6–8]
**6**	Quercetin-3-O-rutinoside (rutin)	*S. kali*<br>*S. tetragona*<br>*S. tetrandra*<br>*S. volkensii*<br>*S. inermis*<br>*S. collina*<br>*S. imbricata*<br>*S. grandis*<br>*S. komarovii*<br>*S. vermiculata*<br>*S. soda*	$C_{27}H_{30}O_{16}$	610.5	[1,3–6,8,10,14,15]
**7**	Rhamnetin	*S. kali*	$C_{16}H_{12}O_7$	316.3	[8,9]
**8**	Isorhmnetin	*S. collina*<br>*S. imbricata*<br>*S. komarovii*	$C_{16}H_{12}O_7$	316.3	[1,10,13,15,16]
**9**	Isorhmnetin-3-O-glucoside	*S. kali*<br>*S. grandis*<br>*S. oppositifolia*<br>*S. komarovii*<br>*S. collina*<br>*S. imbricata*<br>*S. tetrandra*<br>*S. inermis*<br>*S. vermiculate*	$C_{22}H_{22}O_{12}$	478.4	[3–5,9,10,13,15–19]

No	Compound Name	Source	Molecular Formula	Molecular weight g/mol	References
**10**	Isorhmnetin-3-O-glucouronide	*S. grandis*<br>*S. soda*	$C_{22}H_{20}O_{13}$	492.4	[3,4,14]
**11**	Isorhmnetin-3-O-galactopyranoside	*S. imbricata*	$C_{22}H_{22}O_{12}$	478	[16]
**12**	Isorhmnetin-7-O-glucoside	*S. collina*	$C_{22}H_{22}O_{12}$	478.4	[19]
**13**	Isorhmnetin-3-O-rutinoside (Narcissin)	*S. kali*<br>*S. grandis*<br>*S. oppositifolia*<br>*S. collina*<br>*S. vermiculata*<br>*S. komarovii*<br>*S. tetrandra*<br>*S. soda*	$C_{28}H_{32}O_{16}$	624.5	[3–5,9,13–15,18]
**14**	Isorhmnetin-3-O-*B*-D-glucuronyl(1'''→4'') *B*-D-glucuronic acid	*S. imbricata*	-	-	[16]
**15**	Isorhmnetin-3-O-*B*-D-diglucuronate dimethyl ester	*S. imbricata*	-	-	[16]
**16**	Isorhamnetin-3-O-α-L-arabinopyranosyl(1→6)- β-D-glucopyranoside	*S. collina*	$C_{26}H_{29}O_{16}$	597	[19]
**17**	Manghaslin	*S. grandis*	$C_{33}H_{40}O_{20}$	756.7	[3,4]
**18**	Kaempferol	*S. kail*<br>*S. longifolia*<br>*S. tetrandra*<br>*S. inermis*<br>*S. collina*<br>*S. baryosma*<br>*S. vermiculata*	$C_{15}H_{10}O_{6}$	286.2	[2,8,10,14]
**19**	Kaempferol-3-methyl ether	*S. inermis*	$C_{16}H_{12}O_{6}$	300.3	[17]
**20**	Kaempferol-3-O- glucoside (astragalin)	*S. komarovii*<br>*S. tetragona*<br>*S. inermis*	$C_{21}H_{20}O_{11}$	448.4	[12,15,17]
**21**	Kaempferol-3-O- rutinoside	*S. komarovii*	$C_{27}H_{30}O_{15}$	594.5	[15]
**22**	Tiliroside	*S. grandis*	$C_{30}H_{26}O_{13}$	594.5	[3,4]
**23**	Salcolin A	*S. collina*	$C_{27}H_{26}O_{11}$	526.5	[20]
**24**	Salcolin B	*S. collina*	$C_{27}H_{26}O_{11}$	526.5	[20]
**25**	Apigenin	*S. imbricata*	$C_{15}H_{10}O_{5}$	270.2	[6]
**26**	Tricin	*S. collina*	$C_{17}H_{14}O_{7}$	330.3	[1,13,19]
**27**	Selagin	*S. collina*	$C_{16}H_{12}O_{7}$	316.3	[19]
**28**	Tricin-7-O-glucoside	*S. collina*	$C_{23}H_{24}O_{12}$	492.4	[1,13,19]
**29**	Tricin-4'-O- β-D-apioside	*S. collina*	$C_{43}H_{42}O_{21}$	894	[19]
**30**	Chrysoeriol-7-O-*B*-D-glucopyranoside	*S. baryosma*	$C_{22}H_{22}O_{11}$	462.4	[21]
**31**	Chrysin	*S. imbricata*	$C_{15}H_{10}O_{4}$	254.2	[6]
**32**	5,3'-Dihydroxy-7,8,2'-	*S. somalensis*	$C_{18}H_{16}O_{7}$	344	[22]

No	Compound Name	Source	Molecular Formula	Molecular weight g/mol	References
	trimethoxy isoflavone				
**33**	5,3'-Dihydroxy-2'-methoxy-6,7-methylenedioxyisoflavone	*S. somalensis*	$C_{17}H_{12}O_7$	328	[22]
**34**	5,3'-Dihydroxy-6,7,8,2'-tetramethoxyisoflavone	*S. somalensis*	$C_{19}H_{18}O_8$	374	[22]
**35**	5,3'-Dihydroxy-6,7,2'-trimethoxyisoflavone	*S. somalensis*	$C_{18}H_{16}O_7$	344	[23]
**36**	5,8,3'-Trihydroxy-7,2'-dimethoxyisoflavone	*S. somalensis*	$C_{17}H_{14}O_7$	330	[23]
**37**	8,3'-Dihydroxy-5,7,2'-trimethoxyisoflavone	*S. somalensis*	$C_{18}H_{16}O_7$	344	[23]
**38**	5,6,3'-Trihydroxy-7,2'dimethoxyisoflavone	*S. somalensis*	$C_{17}H_{14}O_7$	330	[23]
**39**	6,7,3'-Trihydroxy5,2'-dimethoxyisoflavone	*S. somalensis*	$C_{17}H_{14}O_7$	330	[23]
**40**	5,6,3'-Trihydroxy-2'-methoxy-7,8-methylenedioxy isoflavone OR 5,8,3'-Trihydroxy-2'-methoxy-6,7-methylendioxyisoflavone	*S. somalensis*	$C_{17}H_{12}O_8$	344	[23]
**41**	3'-Hydroxy-5,6,7,2'-tetramethoxyisoflavone	*S. somalensis*	$C_{19}H_{18}O_7$	358	[23]
**42**	7,3'-Dihydroxy-5,6,2'-trimethoxyisoflavone	*S. somalensis*	$C_{18}H_{16}O_7$	344	[23]
**43**	6,3'-Dihydroxy-5,7,2'-trimethoxyisoflavone	*S. somalensis*	$C_{18}H_{16}O_7$	344	[23]
**44**	5,7,8,2',3'-Pentamethoxyisoflavone	*S. somalensis*	$C_{20}H_{20}O_7$	372	[22]
**45**	5,2',3'-Trimethoxy-6,7-methylendioxyisoflavone	*S. somalensis*	-	-	[22]
**46**	Salisoflavan	*S. imbricata*	$C_{17}H_{18}O_6$	318.1	[24]
**47**	5,2'-Dihydroxy-6,7-methylenedioxyisoflavone	*S. collina*	$C_{16}H_{10}O_6$	298	[1]
**48**	Tetranin B	*S. tetrandra*	$C_{17}H_{12}O_7$	329.1	[25]
**49**	Naringenin	*S. imbricata*	$C_{15}H_{12}O_5$	272.3	[6]
**50**	Hesperetin	*S. imbricata*	$C_{16}H_{14}O_6$	302.3	[6]
**51**	Hesperidin	*S. imbricata*	$C_{28}H_{34}O_{15}$	610.6	[6]
**52**	Catechin	*S. imbricata*	$C_{15}H_{14}O_6$	290.3	[6]
**53**	(-) Epicatechin	In the most of the *Salsola* species except *kali* and *tetragona*	$C_{15}H_{14}O_6$	290.3	[8]

Table S2: List of phenolic compounds isolated from different *Salsola* species

No	Compound Name	Source	Molecular Formula	Molecular weight g/mol	References
**54**	Hydroxy tyrosol-4′-glucopyranoside (Biphenol 2)	*S. komarovii*	$C_{14}H_{24}O_9$	336	[26]
**55**	2-(3,4-dihydroxy)-Phenyl-ethyl-*B*-D-glucopyranoside	*S. komarovii*	-	-	[26]
**56**	Cuneataside C	*S. komarovii*	$C_{19}H_{28}O_{12}$	448.4	[26]
**57**	Benzyl-6-O-*B*-D-apiofuranosyl-*B*-D-glucopyranoside	*S. komarovii*	-	-	[26]
**58**	Catechol	*S. imbricata*	$C_6H_6O_2$	110.1	[6]
**59**	Tetranin A	*S. tetrandra*	$C_{15}H_{16}O_4$	260	[25]
**60**	Biphenylsalsinol	*S. villosa*	$C_{20}H_{22}O_6$	381	[27]
**61**	Biphenylsalsonoid B	*S. imbricata*	$C_{21}H_{24}O_8$	404	[28]
**62**	Biphenylsalsonoid A	*S. imbricata*	$C_{20}H_{22}O_7$	374	[28]
**63**	Acanthoside D	*S. collina*	$C_{34}H_{46}O_{18}$	742.7	[19]
**64**	Salsolide	*S. baryosma*	$C_{32}H_{32}O_{11}$	591.2	[29]
**65**	Phloroglucin	*S. tetrandra*<br>*S. tetragona*<br>*S. volkensii*	$C_6H_6O_3$	126	[8]
**66**	3,4,5-Trimethoxyphenyl-B-D-glucopyranoside	*S. tetrandra*	$C_{15}H_{22}O_9$	346	[30]
**67**	Canthoside C	*S. tetragona*<br>*S. komarovii*	$C_{18}H_{26}O_{12}$	434.4	[12,26]
**68**	Canthoside D	*S. tetragona*	$C_{18}H_{26}O_{12}$	434.1	[12]
**69**	Tachioside	*S. komarovii*	$C_{13}H_{18}O_8$	302.3	[26]
**70**	Isotachioside	*S. komarovii*	$C_{13}H_{18}O_8$	302.3	[26]

Table S3: List of phenolic acids isolated from different *Salsola* species

No	Compound Name	Source	Molecular Formula	Molecular weight g/mol	References
**71**	*p*-Hydroxybenzoic acid	*S. kali*<br>*S. collina*<br>*S. imbricata*	$C_7H_6O_3$	138.1	[1,16,31,32]
**72**	Salicylic acid	*S. collina*<br>*S. imbricate*<br>*S. vermiculata*	$C_7H_6O_3$	138.1	[1,6,19,32]
**73**	Anisic acid	*S. collina*	$C_8H_8O_3$	152.1	[1]
**74**	Benzoic acid	*S. imbricata*	$C_7H_6O_2$	122.1	[6]
**75**	Protocatechuic acid	*S. kali*<br>*S. longifolia*<br>*S. tetragona*<br>*S. collina*<br>*S. imbricate*<br>*S. vermiculata*	$C_7H_6O_4$	154.1	[6,8,31,32]
**76**	Gallic acid	*S. kali*<br>*S. imbricate*<br>*S. vermiculata*	$C_7H_6O_5$	170.1	[6,8]
**77**	Syringic acid	*S. kali*<br>*S. collina*	$C_9H_{10}O_5$	198.2	[31,32]
**78**	Vanillic acid	*S. kali*<br>*S. tetragona*<br>*S. collina*<br>*S. imbricate*<br>*S. vermiculata*	$C_8H_8O_4$	168.1	[6,12,31,32]
**79**	Protocatechuic aldehyde	*S. collina*	$C_7H_6O_3$	138.1	[1]
**80**	α- resorcyclic	*S. kali*	$C_7H_6O_4$	154.1	[31]
**81**	β-resorcyclic	*S. kali*	$C_7H_6O_4$	154.1	[31]
**82**	Gentisic acid	*S. kali*<br>*S. longifolia*	$C_7H_6O_4$	154.1	[8,31]
**83**	Isovanillic acid	*S. imbricata*	$C_8H_8O_4$	168.1	[16]
**84**	Hypogallic acid	*S. kali*<br>*S. tetrandra*<br>*S. volkensii*<br>*S. inermis*	$C_7H_6O_4$	154.1	[8]
**85**	Orsellic acid	*S. collina*	$C_8H_8O_4$	168.1	[32]
**86**	*p*-Hydroxyphenylacetic	*S. kali*	$C_8H_8O_3$	152.1	[31]
**87**	Rosmarinic acid	*S. imbricate*<br>*S. vermiculata*	$C_{18}H_{16}O_8$	360.3	[6,7]
**88**	*p*-Hydroxycinnamic acid	*S. collina*<br>*S. kali*<br>*S. imbricata*	$C_9H_8O_3$	164.2	[1,6,19,31–33]
**89**	Caffeic acid	*S. kali*<br>*S. collina*<br>*S. imbricate*<br>*S. vermiculata*	$C_9H_8O_4$	180.2	[6,11,31,32]

No	Compound Name	Source	Molecular Formula	Molecular weight g/mol	References
**90**	Ferulic acid	*S. kali*<br>*S. collina*<br>*S. imbricate*<br>*S. vermiculata*	$C_{10}H_{10}O_4$	194.2	[6,11,16,19,31,32]
**91**	Cinnamic acid	*S. imbricate*<br>*S. vermiculata*	$C_9H_8O_2$	148.2	[6]
**92**	Acetyl ferulic acid	*S. collina*	$C_{12}H_{12}O_5$	236.2	[1]
**93**	Chlorogenic acid	*S. imbricata*<br>*S. kali*	$C_{16}H_{18}O_9$	354.3	[6,11]
**94**	Neochlorogenic acid	*S. kali*	$C_{16}H_{18}O_9$	354.3	[11]
**95**	Isochlorogenic acid	*S. kali*	$C_{25}H_{24}O_{12}$	516.4	[11]

Table S4: List of nitrogenous compounds isolated from different *Salsola* species

No	Compound Name	Source	Molecular Formula	Molecular weight g/mol	References
**96**	Moupinamide	*S. collina*	$C_{18}H_{19}NO_4$	313.3	[1]
**97**	2'-Hydroxymoupinamide	*S. collina*	$C_{18}H_{21}NO_5$	331	[1]
**98**	2'-Hydroxy- 3''-methylmoupinamide	*S. collina*	$C_{19}H_{23}NO_6$	361	[1]
**99**	*N*-[2′-(3′′,4′′-Dihydroxyphenyl)-2′-hydroxyethyl]-3-(4′′′-methoxyphenyl)prop-2-enamide	*S. foetida*	$C_{18}H_{19}NO_5$	329	[34]
**100**	*N*-[2′-(3′′,4′′-Dihydroxyphenyl)-2′-hydroxyethyl]-3-(3′′′,4′′′-dimethoxyphenyl)prop-2-enamide	*S. foetida*	$C_{19}H_{21}NO_6$	359	[34]
**101**	*N*-[2′-(3′′-Hydroxy-4′′-methoxyphenyl)-2′-hydroxyethyl]3-(4′′′-methoxyphenyl)-prop-2-enamide	*S. foetida*	$C_{19}H_{21}NO_5$	343	[34]
**102**	*N*-trans-feruloyl 3-O-methyldopamine	*S. collina*<br>*S. komarovii*	$C_{19}H_{21}NO_5$	343	[15,33,35]
**103**	*N*-trans-feruloyl tyramine	*S. komarovii*<br>*S. tetrandra*<br>*S. imbricata*<br>*S. vermiculata*<br>*S. tetrandra*	$C_{18}H_{19}NO_4$	313.3	[5,15,16,30]
**104**	*trans-N*-feruloyltyramine-4'''-O-B-D-glucopyranoside	*S. inermis*	$C_{24}H_{30}NO_9$	476.3	[17]
**105**	*S-(-)-trans-N*-feruloyloctopamine	*S. tetrandra*	$C_{18}H_{19}NO_5$	329.3	[30]
**106**	*N-*Caffeoyltyramine	*S. vermiculata*<br>*S. tetrandra*	$C_{17}H_{17}NO_4$	299.3	[5]
**107**	*N*-Feruloyl- 3'''-methoxytyramine	*S. vermiculata*<br>*S. tetrandra*	$C_{19}H_{21}NO_5$	343.3	[5]

No	Compound Name	Source	Molecular Formula	Molecular weight g/mol	References
**108**	*N*-(3',4'-Dimethoxy-cinnamoyl)-norepinephrine	*S. vermiculata*<br>*S. tetrandra*	$C_{19}H_{21}NO_6$	359.3	[5]
**109**	*N*-(4'-Methoxy-cinnamoyl)-norepinephrine	*S. vermiculata*<br>*S. tetrandra*	$C_{18}H_{19}NO_5$	329.3	[5]
**110**	Salsoline	*S. collina*<br>*S. kali (S. tragus)*<br>*S. soda*<br>*S. oppositifolia*<br>*S. vermiculata*	$C_{11}H_{15}NO_2$	193.2	[18,36–38]
**111**	*N*-Methylisosalsolidine	*S. tragus*<br>*S. soda*<br>*S. oppositifolia*	$C_{12}H_{17}NO_2$	207.3	[38]
**112**	Salsolidine	*S. collina*<br>*S. kali (S. tragus)*<br>*S. soda*<br>*S. oppositifolia*<br>*S. vermiculata*	$C_{12}H_{17}NO_2$	207.3	[18,36–38]
**113**	Carnegine	*S. soda*<br>*S. kali*<br>*S. oppositifolia*	$C_{13}H_{19}NO_2$	221.3	[37,38]
**114**	Salsoline A	*S. tetrandra*<br>*S. vermiculata*<br>*S. collina*	$C_{12}H_{13}NO_3$	219	[1,5,33,35]
**115**	Salsoline B	*S. collina*	$C_{12}H_{13}NO_3$	219	[33]
**116**	*N*-(4-methylpentanoyl) tyramine	*S. vermiculata*	$C_{14}H_{21}NO_2$	235	[5]
**117**	Uracil	*S. collina*	$C_4H_4N_2O_2$	112.1	[1]
**118**	Uridine	*S. collina*	$C_9H_{12}N_2O_6$	244.2	[1]
**119**	Terrestric acid	*S. collina*	$C_4H_5N_4O_3$	157	[1]
**120**	Pericampylinone-A	*S. collina*	$C_9H_9NO_3$	179.2	[1]
**121**	*N*-Acetyltryptophan	*S. collina*<br>*S. grandis*	$C_{13}H_{14}N_2O_3$	246.3	[1,3,4]
**122**	Betaine	*S. rigida*<br>*S. longifolia*<br>*S. kali*<br>*S. tetrandra*	$C_5H_{11}NO_2$	117.2	[39]
**123**	Methyl carbamate	*S. rigida*<br>*S. longifolia*<br>*S. kali*<br>*S. tetrandra*	$C_2H_5NO_2$	75.1	[40]
**124**	Salisomide	*S. imbricata*	$C_{38}H_{69}NO_2$	571.5	[24]
**125**	Triacetonamine	*S. kali*<br>*S. tetrandra*<br>*S. rigida*<br>*S. longifolia*	$C_9H_{17}NO$	155.2	[39]
**126**	Tridecanamine	*S. tetrandra*	$C_{13}H_{29}N$	199.4	[41]

Table S5: List of saponin compounds isolated from different *Salsola* species

No	Compound Name	Source	Molecular Formula	Molecular weight g/mol	References
**127**	Momordin II b	*S. grandis*	$C_{42}H_{66}O_{14}$	794	[3,4,42]
**128**	Momordin II c	*S. grandis*<br>*S. soda*	$C_{47}H_{74}O_{18}$	926	[3,4]
**129**	Pseudoginseoside RT1	*S. imbricata*	$C_{47}H_{74}O_{18}$	926	[42]
**130**	Salsoloside C	*S. micranthera*	$C_{47}H_{74}O_{18}$	926	[43,44]
**131**	Salsoloside D	*S. micranthera*	$C_{47}H_{74}O_{19}$	942	[43,44]
**132**	Salsoloside E	*S. micranthera*	$C_{53}H_{84}O_{23}$	1088	[43,44]
**133**	Olean-12-en-28-oic acid (Oleanolic acid)	*S. inermis*	$C_{30}H_{48}O_{3}$	456	[17]
**134**	Olean-12-en-3,28-diol	*S. inermis*	$C_{30}H_{50}O_{2}$	442	[17]
**135**	Boussingoside A2	*S. imbricata*	$C_{41}H_{62}O_{14}$	778	[42]
**136**	3-O-*B*-D-xylopyranosyl-(1 - 2)-O- *B*-D-glucuronopyranosyl-akebonic acid-28-O-*B*-D-glucopyranoside	*S. imbricata*	$C_{46}H_{70}O_{18}$	910	[42]
**137**	3-O-*B*-D-xylopyranosyl-(1-2)-O-*B*-D-glucuronopyranosyl-29-hydroxyoleanolic acid-28-O-*B*-D-glucopyranoside	*S. imbricata*	$C_{47}H_{74}O_{19}$	942	[42]

Table S6: List of triterpenes isolated from different *Salsola* species

No	Compound Name	Source	Molecular Formula	Molecular weight g/mol	References
**138**	Corchoionoside C	*S. collina*	$C_{19}H_{30}O_{8}$	386.4	[1]
**139**	Lupeol	*S. kali*	$C_{30}H_{50}O$	426.7	[45]
**140**	Salsolic acid	*S. baryosma*	$C_{30}H_{48}O_{5}$	488.7	[46]
**141**	Ursolic acid	*S. kali*	$C_{30}H_{48}O_{3}$	456.7	[45]
**142**	Salsolin A	*S. baryosma*	$C_{30}H_{48}O_{6}$	504	[46]
**143**	Salsolin B	*S. baryosma*	$C_{36}H_{59}O_{12}$	683	[46]
**144**	$2\alpha,3\beta$,23,24-tetrahydroxyurs-12-en-28-oic acid	*S. baryosma*	$C_{30}H_{48}O_{6}$	504	[46]

Table S7: List of sterols isolated from different *Salsola* species

No	Compound Name	Source	Molecular Formula	Molecular weight g/mol	References
**145**	Sitostanol = Stigmastanol	*S. collina*<br>*S. kali*<br>*S. tetrandra*<br>*S. rigida*<br>*S. longifolia*<br>*S. inermis*	$C_{29}H_{52}O$	416.7	[10,13]
**146**	Stigmasterol	*S. kali*<br>*S. tetrandra*<br>*S. rigida*<br>*S. longifolia*<br>*S. inermis*<br>*S. collina*	$C_{29}H_{48}O$	412	[10,13,17,47,48]
**147**	Stigmasterol- 3-*B*-O-D-glucopyranoside	*S. inermis*	$C_{35}H_{58}O_6$	574.8	[17]
**148**	Avenasterol	*S. kali*<br>*S. tetrandra*<br>*S. rigida*<br>*S. longifolia*	$C_{29}H_{48}O$	412.7	[47,48]
**149**	*B*- Sitosterol	*S. kali*<br>*S. inermis*<br>*S. collina*<br>*S. oppositifolia*	$C_{29}H_{50}O$	414.7	[10,17,18,45]
**150**	*B*- Sitosterol-3-O-glucoside	*S. kali*	$C_{35}H_{60}O_6$	576.8	[49]
**151**	Campesterol	*S. collina*	$C_{28}H_{48}O$	400.7	[10,13]

Table S8: List of fatty acids isolated from different *Salsola* species

No	Compound Name	Source	Molecular Formula	Molecular weight g/mol	References
**152**	2- Monolinolenin	*S. oppositifolia*	$C_{21}H_{36}O_4$	352.5	[18]
**153**	2,3 Dihydroxy propyl palmitate	*S. tetragona*	$C_{19}H_{38}O_4$	330.5	[12]
**154**	8- Hexadecynoic acid (stearolic acid)	*S. tetrandra*	$C_{18}H_{32}O_2$	280.4	[41]
**155**	9,12,13 Trihydroxy octadeca 7-enoic acid	*S. vermiculata*<br>*S. tetrandra*	$C_{18}H_{34}O_5$	330.5	[5]
**156**	9,12,13 Trihydroxy decosan 10,15,19-trienoic acid	*S. inermis*	$C_{22}H_{42}O_5$	386	[17]
**157**	9,12,13 Trihydroxy octadeca-10(*E*),15(*Z*)-dienoic acid	*S. tetrandra*	$C_{18}H_{32}O_5$	328.4	[30]
**158**	9,12,13 Trihydroxy octadeca-10(*E*)-dienoic acid	*S. tetrandra*	$C_{18}H_{34}O_5$	330.2	[30]
**159**	9,12 Octadecadienoic (*Z,Z*), methyl ester (methyl linoleate)	*S. tetrandra*<br>*S. oppositifolia*	$C_{19}H_{34}O_2$	294.5	[18,41]
**160**	11- Eicosenoic	*S. tetrandra*	$C_{20}H_{38}O_2$	310.5	[41]
**161**	Arachidic acid	*S. tetrandra*<br>*S. vermiculata*	$C_{20}H_{40}O_2$	312.5	[41]
**162**	Arachidonic acid	*S. kali*	$C_{20}H_{32}O_2$	304.5	[47]
**163**	Behenic acid (docosanoic)	*S. tetrandra*	$C_{22}H_{44}O_2$	340.6	[41]
**164**	Hexacosanoic acid	*S. tetrandra*	$C_{26}H_{52}O_2$	396.7	[41]
**165**	Hydoxy octadecatrienoic acid	*S. vermiculata*<br>*S. tetrandra*	$C_{18}H_{30}O_3$	294.4	[5]
**166**	Hydoxy octadecadienoic acid	*S. vermiculata*<br>*S. tetrandra*	$C_{18}H_{32}O_3$	296.4	[5]
**167**	Lauric acid	*S. tetrandra*	$C_{12}H_{24}O_2$	200.3	[41]
**168**	Lignoceric acid (tetracosanoic)	*S. tetrandra*<br>*S. vermiculate*	$C_{24}H_{48}O_2$	368.6	[41]
**169**	Nonadecanoic acid	*S. tetrandra*	$C_{19}H_{38}O_2$	298	[41]
**170**	Linoleic acid	*S. tetrandra*<br>*S. collina*<br>*S. vermiculate*<br>*S. oppositifolia*	$C_{18}H_{32}O_2$	280.5	[5,10,18,41]
**171**	Methyl linolenate	*S. oppositifolia*	$C_{19}H_{32}O_2$	292.5	[18]
**172**	Linolenic acid	*S. tetrandra*<br>*S. collina*<br>*S. vermiculate*<br>*S. kali*	$C_{18}H_{30}O_2$	278.4	[5,10,41,47]
**173**	Methyl palmitate	*S. oppositifolia*<br>*S. tetrandra*	$C_{17}H_{34}O_2$	270.5	[18,41]
**174**	Heptadecanoic (margaric) acid	*S. tetrandra*	$C_{17}H_{34}O_2$	270.5	[41]
**175**	Myristic acid	*S. tetrandra*	$C_{14}H_{28}O_2$	228.4	[41]
**176**	Methyl stearate	*S. oppositifolia*	$C_{19}H_{38}O_2$	298.5	[18]

No	Compound Name	Source	Molecular Formula	Molecular weight g/mol	References
**177**	Octacosanoic acid	*S. tetrandra*	$C_{28}H_{56}O_2$	424.7	[41]
**178**	Oleic acid	*S. kali*<br>*S. tetrandra*<br>*S. tetragona*<br>*S. collina*<br>*S. vermiculate*	$C_{18}H_{34}O_2$	282.5	[5,10,12,41,47]
**179**	Octadecanoic acid, 2,3-dihydroxypropyl ester (monostearin)	*S. tetrandra*	$C_{21}H_{42}O_4$	358.6	[41]
**180**	Palmitic acid	*S. tetrandra*<br>*S. collina*<br>*S. vermiculate*<br>*S. oppositifolia*<br>*S. kali*	$C_{16}H_{32}O_2$	256.4	[5,10,18,41,47]
**181**	Palmitoleic acid	*S. tetrandra*	$C_{16}H_{30}O_2$	254.4	[41]
**182**	Stearic acid	*S. tetrandra*<br>*S. kali*<br>*S. vermiculate*	$C_{18}H_{36}O_2$	284.5	[41,47]
**183**	Trihydroxy octadecadienoic acid	*S. tetrandra*<br>*S. vermiculate*	$C_{18}H_{32}O_5$	328.4	[5]
**184**	Tetradecanoic acid, methyl ester (myristic acid, methyl ester)	*S. tetrandra*	$C_{15}H_{30}O_2$	242.4	[41]
**185**	Tricosanoic acid	*S. tetrandra*	$C_{23}H_{46}O_2$	354.6	[41]
**186**	*cis*-10- Heptadecanoic acid	*S. tetrandra*	$C_{17}H_{32}O_2$	268	[41]

Table S9: List of volatile constituents isolated from different *Salsola* species

No	Compound Name	Source	Molecular Formula	Molecular weight g/mol	References
**187**	Carvone	*S. vermiculata*	$C_{10}H_{14}O$	150.2	[50],
**188**	Linalool	*S. vermiculata*	$C_{10}H_{18}O$	154.3	[50]
**189**	9- Hydroxylinaloyl glucoside	*S. tetrandra*	$C_{16}H_{28}O_7Na$	355.2	[30]
**190**	Limonene	*S. vermiculata*	$C_{10}H_{16}$	136.2	[50]
**191**	*Β*-Caryophyllene	*S. vermiculata*	$C_{15}H_{24}$	204.4	[50]
**192**	Vanillin	*S. collina*	$C_8H_8O_3$	152.2	[1]
**193**	Cumin aldehyde	*S. vermiculata*	$C_{10}H_{12}O$	148.2	[50]
**194**	Benzyl salicylate	*S. cyclophylla*	$C_{14}H_{12}O_3$	228	[51]
**195**	Hexa hydrofarnesyl acetone	*S. cyclophylla*	$C_{18}H_{36}O$	268	[51]

Table S10: List of lignanas isolated from different *Salsola* species

No	Compound Name	Source	Molecular Formula	Molecular weight g/mol	References
**196**	(+)-Lyoniresinol 9'-*O*-*β*-D - glucopyranoside	*S. komarovii*	$C_{28}H_{38}O_{13}$	582.6	[26]
**197**	(8*S*,8'*R*,7'*R*)-9'-[(Β-glucopyranosyl)oxy] lyoniresinol	*S. komarovii*	-	-	[26]
**198**	Lariciresinol-9´-O-**β**-D-glucopyranoside	*S. komarovii*	$C_{38}H_{46}O_{17}$	774.8	[26]
**199**	Alangilignoside C	*S. komarovii*	$C_{28}H_{38}O_{13}$	582	[26]
**200**	Conicaoside	*S. komarovii*	$C_{27}H_{36}O_{12}$	552	[26]

Table S11: List of magastigmane isolated from different *Salsola* species

No	Compound Name	Source	Molecular Formula	Molecular weight g/mol	References
**201**	Icariside B_2	*S. komarovii*	$C_{19}H_{30}O_8$	386.4	[26]
**202**	Staphylionoside D	*S. komarovii*	$C_{19}H_{30}O_8$	386.4	[26]
**203**	Blumenyl A *β*-D-glucopyranoside	*S. komarovii*	-	-	[26]
**204**	(6*R*,9*S*)-3-oxo-*α*-ionol *β*-D-glucopyranoside	*S. komarovii*	-	-	[26]
**205**	3-oxo-*α*-ionol 9-*O*-*β*-D-apiofuranosyl-(1→6)-*β*-D-glucopyranoside	*S. komarovii*	-	-	[26]
**206**	Blumenyl B *β*-D-glucopyranoside	*S. komarovii*	$C_{19}H_{32}O_8$	388	[26]
**207**	Blumenol B 9-*O*-*β*-D-apiofuranosyl-(1→6)-*β*-D-glucopyranoside	*S. komarovii*	-	-	[26]

Table S12: List of coumarins isolated from different *Salsola* species

No	Compound Name	Source	Molecular Formula	Molecular weight g/mol	References
**208**	Daphnoretin	*S. baryosma*	$C_{19}H_{12}O_7$	352.3	[21]
**209**	Daphnorin	*S. baryosma*	$C_{25}H_{22}O_{12}$	514.4	[21]
**210**	Umbelliferone	*S. inermis*	$C_9H_6O_3$	162.1	[17]
**211**	Scopoletin	*S. baryosma* *S. inermis*	$C_{10}H_8O_4$	192.2	[17,21]
**212**	Scopoletin 7-O-*B*-D-glucopyranside	*S. laricifolia*	$C_{16}H_{18}O_9$	354.3	[52]
**213**	Fraxidin	*S. laricifolia*	$C_{11}H_{10}O_5$	222.2	[52]
**214**	Isofraxidin	*S. laricifolia*	$C_{11}H_{10}O_5$	222.2	[52]
**215**	Fraxetin	*S. laricifolia*	$C_{10}H_8O_5$	208.2	[52]
**216**	Isofraxidin 7-O-*B*-D-glucopyranoside (calycanthoside)	*S. laricifolia*	$C_{17}H_{20}O_{10}$	384.3	[52]
**217**	Fraxidin-8-O-*B*-D-glucopyranoside	*S. laricifolia*	$C_{17}H_{20}O_{10}$	384.3	[52]
**218**	Bergaptol	*S. baryosma*	$C_{11}H_6O_4$	202.2	[21]
**219**	Bergaptol-5-O-*B*-D-glucopyranoside	*S. baryosma*	$C_{17}H_{17}O_9$	365	[21]

Table S13: List of cardiac glycosides isolated from different *Salsola* species

No	Compound Name	Source	Molecular Formula	Molecular weight g/mol	References
**220**	3-O-*B*-D-Allopyranosyl-coroglaucigenin (salsotetragonin)	*S. tetragona*	$C_{29}H_{44}O_{10}$	553.3	[12]
**221**	Uzarigenin	*S. tetragona*	$C_{23}H_{34}O_4$	374.5	[12]
**222**	Desglucouzarin	*S. tetragona*	$C_{29}H_{44}O_9$	536.7	[12]
**223**	12-Dehydroxyghalakinoside	*S. tetragona*	$C_{29}H_{42}O_{10}$	551.3	[12]
**224**	Calactin	*S. tetragona*	$C_{29}H_{40}O_9$	532.6	[12]

Table S14: List of alcohols isolated from different *Salsola* species

No	Compound Name	Source	Molecular Formula	Molecular weight g/mol	References
**225**	Salsolanol	*S. villosa*	$C_{12}H_{18}O_3$	210	[27]
**226**	Phytol	*S. oppositifolia*	$C_{20}H_{40}O$	296	[18]
**227**	3,9 Diethyl-6-tridecanol	*S. tetrandra*	$C_{17}H_{36}O$	256	[41]
**228**	2,7-Dimethyl-1-octanol	*S. tetrandra*	$C_{10}H_{22}O$	158	[41]

Table S15: List of cyanogenic, isoprenoid, and Sulphur containing compounds isolated from different *Salsola* species

No	Compound Name	Source	Molecular Formula	Molecular weight g/mol	References
**229**	Taxiphllin	*S. tetrandra*	$C_{14}H_{17}NO_7$	311	[30]
**230**	3-*β*-hydroxy-5α,6α-epoxy-*β*-ionone-2-α-*O*-*β*-D-glucopyranoside (Norisoprenoid)	*S. tetrandra*	$C_{19}H_{30}O_9$	402	[30]
**231**	Isohexyl 2- pentyl ester, sulfurous acid	*S. tetrandra*	$C_{11}H_{24}O_3S$	236	[41]

References

1. Jin, Y.-S.; Du, J.-L.; Yang, Y.; Jin, L.; Song, Y.; Zhang, W.; Chen, H.-S. Chemical and biologically active constituents of *Salsola collina*. *Chem. Nat. Compd.* **2011**, *47*, 257-260.
2. Kaur, P.; Bains, N. Extraction of flavonoids from in vitro and in vivo tissue culture of some important halophytes of western Rajasthan. *Int. J. Pharmtech Res.* **2012**, *4*, 1167-1171.
3. Orhan, I.E.; Kucukboyaci, N.; Calis, I.; Cerón-Carrasco, J.P.; den-Haan, H.; Peña-García, J.; Pérez-Sánchez, H. Acetylcholinesterase inhibitory assessment of isolated constituents from *Salsola grandis* Freitag, Vural & Adıgüzel and molecular modeling studies on N-acetyltryptophan. *Phytochem. Lett.* **2017**, *20*, 373-378.
4. Küçükboyacı, N.; Küpeli Akkol, E.; Suntarİhsan Çalış, İ.; Çalış, İ. In vivo Anti-Inflammatory and Antinociceptive Activities of the Extracts and Chemical Constituents of an Endemic Turkish Plant, *Salsola grandis*. *Rec. Nat. Prod.* **2016**, *10*.
5. Rasheed, D.M.; El Zalabani, S.M.; Koheil, M.A.; El-Hefnawy, H.M.; Farag, M.A. Metabolite profiling driven analysis of *Salsola* species and their anti-acetylcholinesterase potential. *Natural Product Research* **2013**, *27*, 2320-2327, doi:10.1080/14786419.2013.832676.
6. Shehab, N.G.; Abu-Gharbieh, E. Phenolic profiling and evaluation of contraceptive effect of the ethanolic extract of *Salsola imbricata* Forssk. in male albino rats. *Evid. Based Complement. Alternat. Med.* **2014**, *2014*, 1-8.
7. Shehab, N.G.; Abu-Gharbieh, E.; Bayoumi, F.A. Impact of phenolic composition on hepatoprotective and antioxidant effects of four desert medicinal plants. *BMC Complement. Altern. Med.* **2015**, *15*, 401.
8. Taia, W.K.; Shiha, M.A.; Al-Kogali, A.A.; Abd-Almaged, A.M. Anatomical and chemo-taxonomical investigations within some *Salsola* L. species grown in the western costal region of Egypt. *Int. J. Sci. Res. Manag.* **2018**, *6*.
9. Tundis, R.; Loizzo, M.; Statti, G.; Menichini, F. Inhibitory effects on the digestive enzyme α-amylase of three *Salsola* species (Chenopodiaceae) in vitro. *Pharmazie* **2007**, *62*, 473-475.

10. Zaikov, K.; Syrchina, A.; Vereshchagin, A.; Chernousova, A.; Semenov, A. An investigation of the chemical composition of the seeds of *Salsola collina*. *Chem. Nat. Compd.* **1992**, *28*, 627-628.
11. Lodhi, M. Allelopathic potential of *Salsola kali* L. and its possible role in rapid disappearance of weedy stage during revegetation. *J. Chem. Ecol.* **1979**, *5*, 429-437.
12. Ghorab, H.; Khettaf, A.; Lehbili, M.; Kabouche, A.; Magid, A.A.; Harakat, D.; Voutquenne-Nazabadioko, L.; Kabouche, Z. A new cardenolide and other compounds from *Salsola tetragona*. *Nat. Prod. Commun.* **2017**, *12*, 1934578X1701200102.
13. Syrchina, A.; Vereshchagin, A.; Larin, M.; Semenov, A. Flavonoids of *Salsola collina*. *Chem. Nat. Compd.* **1989**, *25*, 619-620.
14. Iannuzzi, A.M.; Moschini, R.; De Leo, M.; Pineschi, C.; Balestri, F.; Cappiello, M.; Braca, A.; Del-Corso, A. Chemical profile and nutraceutical features of *Salsola soda* (agretti): Anti-inflammatory and antidiabetic potential of its flavonoids. *Food Bioscience* **2020**, *37*, 100713.
15. Lee, H.J.; Pan, C.-H.; Kim, E.-S.; Kim, C.Y. Online high performance liquid chromatography (HPLC)-ABTS+ based assay and HPLC-electrospray ionization mass spectrometry analysis of antioxidant phenolic compounds in *Salsola komarovii*. *J. Korean Soc .Appl. Biol. Chem.* **2012**, *55*, 317-321.
16. Osman, S.M.; El Kashak, W.A.; Wink, M.; El Raey, M.A. New isorhamnetin derivatives from *Salsola imbricata* Forssk. leaves with distinct anti-inflammatory activity. *Pharmacogn. Mag.* **2016**, *12*, S47.
17. Elsharabasy, F.S.; Hosney, A.M. Chemical constituents from the aerial parts of *Salsola inermis*. *Egypt. Pharm. J.* **2013**, *12*, 90.
18. Tundis, R.; Loizzo, M.R.; Bonesi, M.; Menichini, F.; Statti, G.A.; Menichini, F. In vitro cytotoxic activity of *Salsola oppositifolia* Desf.(Amaranthaceae) in a panel of tumour cell lines. *Z. Naturforsch. C.* **2008**, *63*, 347-354.
19. Xiang, Y.; Li, Y.-B.; Zhang, J.; Li, P.; Yao, Y.-Z. Studies on chemical constituents of *Salsola collina*. *China journal of Chinese materia medica* **2007**, *32*, 409-413.
20. Syrchina, A.; Gorshkov, A.; Shcherbakov, V.; Zinchenko, S.; Vereshchagin, A.; Zaikov, K.; Semenov, A. Flavonolignans of *Salsola collina*. *Chem. Nat. Compd.* **1992**, *28*, 155-158.
21. Ahmad, S.; Maharvi, G.; Ashraf, M.; Riaz, N.; Afza, N.; Khan, K.; Khan, M.; Jabbar, A.; Janbaz, K. Phytochemical studies on *Salsola baryosma*. *J. Chem. Soc. Pak.* **2006**, *28*, 176-178.
22. Woldu, Y.; Abegaz, B. Isoflavonoids from *Salsola somalensis*. *Phytochemistry* **1990**, *29*, 2013-2015.
23. Abegaz, B.M.; Woldu, Y. Isoflavonoids from the roots of *Salsola somalensis*. *Phytochemistry* **1991**, *30*, 1281-1284.
24. Saleem, M.; Akhter, N.; Shaiq Ali, M.; Nazir, M.; Riaz, N.; Moazzam, M.; Arshad, M.; Jabbar, A. Structure determination of salisomide and salisoflavan, two new secondary metabolites from *Salsola imbricata*, by 1D and 2D NMR spectroscopy. *Magn. Reson. Chem.* **2009**, *47*, 263-265.
25. Beyaoui, A.; Chaari, A.; Ghouila, H.; Ali Hamza, M.h.; Ben Jannet, H. New antioxidant bibenzyl derivative and isoflavonoid from the Tunisian *Salsola tetrandra* Folsk. *Nat. Prod. Res.* **2012**, *26*, 235-242.

26. Cho, H.K.; Suh, W.S.; Kim, K.H.; Kim, S.Y.; Lee, K.R. Phytochemical constituents of *Salsola komarovii* and their effects on NGF induction. *Nat. Prod. Sci.* **2014**, *20*, 95-101.
27. Oueslati, M.H.; Al-Ghamdi, F.A.; Noubigh, A. Two new bioactive salsolanol and biphenylsalsinol from the aerial parts of *Salsola villosa* Delile. ex Schul.(Chenopodiaceae) growing in Saudi Arabia. *Asian Pac. J. Trop. Biomed.* **2015**, *5*, 624-628.
28. Oueslati, M.H.; Bouajila, J.; Jannet, H.B. Two New Bioactive Biphenylpropanoidsfrom the Roots of *Salsola*
imbricata (Chenopodiaceae) Growing in Saudi Arabia. *Orient. J. Chem.* **2017**, *33*, 1871-1878.
29. Ahmad, S.; Ashraf, M.; Riaz, N.; Choudhary, M.I.; Maharvi, G.M.; Afza, N.; Jabbar, A.; Janbaz, K.H.; Khan, M.S. Salsolide, a new p-hydroxyphenylglycol derivative from *Salsola baryosma*. *Journal of The Chemical Society of Pakistan* **2008**, *30*, 110-112.
30. Oueslati, M.H.; Ben Jannet, H.; Mighri, Z.; Chriaa, J.; Abreu, P.M. Phytochemical constituents from *Salsola tetrandra*. *J. Nat. Prod.* **2006**, *69*, 1366-1369.
31. Sokolowska-Krzaczek, A.; Skalicka-Wozniak, K.; Czubkowska, K. Variation of phenolic acids from herb and roots of *Salsola kali* L. *Acta Soc. Bot. Pol.* **2009**, *78*, 197-201.
32. Zhao, X.-L.; Yuan, W.; Li, Z.-Z.; Jin, H.; Gong, Y.-L. *Salsola collina* ethyl acetate extract alleviates diabetic gastroparesis possibly through oxidative stress inhibition. In Proceedings of IOP Conference Series: Earth and Environmental Science; p. 012021.
33. Xiang, Y.; Li, Y.-B.; Zhang, J.; Li, P.; Yao, Y.-Z. A new alkaloid from *Salsola collina*. *Acta Pharm. Sin.* **2007**, *42*, 618-620.
34. Khan, K.M.; Maharvi, G.M.; Abbaskhan, A.; Hayat, S.; Khan, M.T.H.; Makhmoor, T.; Choudhary, M.I.; Shaheen, F. Three tyrosinase inhibitors and antioxidant compounds from *Salsola foetida*. *Helv. Chim. Acta* **2003**, *86*, 457-464.
35. Zhao, Y.; Ding, X. Studies on the alkaloids from *Salsola collina* Pall. *Acta Pharm. Sin.* **2004**, *39*, 598-600.
36. Glushchenko, A.; Georgiyants, V.; Bevz, N. Development and Estimation of Validation Characteristics For The Quantitative Determination of Glycoalkaloid in *Salsola collina* L. Extracts. *Acta Chim. Pharm. Indica* **2015**, *5*, 47-54.
37. Loizzo, M.R.; Tundis, R.; Statti, G.A.; Passalacqua, N.G.; Peruzzi, L.; Menichini, F. In vitro angiotensin converting enzyme inhibiting activity of *Salsola oppositifolia* Desf., *Salsola soda* L. and *Salsola tragus* L. *Nat. Prod. Res.* **2007**, *21*, 846-851.
38. Tundis, R.; Menichini, F.; Conforti, F.; Loizzo, M.R.; Bonesi, M.; Statti, G.; Menichini, F. A potential role of alkaloid extracts from *Salsola* species (Chenopodiaceae) in the treatment of Alzheimer's disease. *J. Enzyme Inhib. Med. Chem.* **2009**, *24*, 818-824.
39. Karawya, M.; Wassel, G.; Ruecker, G.; Baghdadi, H.; Ahmed, Z. Isolation of triacetonamine from *Salsola tetrandra*. *Phytochemistry* **1971**, *10*, 3303-3304.
40. Karawya, M.; Wassel, G.; Baghdadi, H.; Ahmed, Z. Isolation of methyl carbamate from four Egyptian *Salsola* species. *Phytochemistry* **1972**.
41. Elsharabasy, F.S.; AL-Mushhin, A.A.; Araffa, S.; Farrag, A.R.H. Phytochemical screening and gastroprotective effect of the aerial parts of *Salasola terrandra* Forssk. Against aspirin induced gastric ulceration in rats. *J. pharmacogn. phytochem.* **2015**, *3*, 221-232.
42. Hamed, A.I.; Masullo, M.; Sheded, M.G.; Mahalel, U.A.; Tawfik, M.M.; Perrone, A.; Piacente, S. Triterpene saponins from *Salsola imbricata*. *Phytochem. Lett.* **2011**, *4*, 353-356.

43. Annaev, C.; Isamukhamedova, M.; Abubakirov, N. Triterpene glycosides of *Salsola micranthera*. I. Structures of salsolosides C and D. *Chem. Nat. Compd.* **1983**, *19*, 691-695.
44. Annaev, C.; Isamukhamedova, M.; Abubakirov, N. Triterpene glycosides of *Salsola micranthera.* II. The strucutre of salsoloside E. *Chem. Nat. Compd.* **1984**, *20*, 60-64.
45. Alturkistani, T.; Bin Afif, M.; Alzahrani, R.; Alnouno, R.; Badr, J.M. Phytochemical investigation of *Salsola kali* extract. *RPBS* **2017**, *1*, 70-72.
46. Ahmad, Z.; Mehmood, S.; Fatima, I.; Malik, A.; Ifzal, R.; Afza, N.; Iqbal, L.; Latif, M.; Nizami, T.A. Structural determination of salsolins A and B, new antioxidant polyoxygenated triterpenes from *Salsola baryosma*, by 1D and 2D NMR spectroscopy. *Magn. Reson. Chem.* **2008**, *46*, 94-98.
47. Karawya, M.; Wassel, G.; Baghdadi, H.; Ahmed, Z. Phytochemical study of certain *Salsola* species. *Planta Med.* **1972**, *21*, 173-176.
48. Salt, T.A.; Adler, J.H. Diversity of sterol composition in the family Chenopodiaceae. *Lipids* **1985**, *20*, 594-601.
49. Aniss, H.A.; Said, A.E.M.; El Sayed, I.H.; Adly, C. Amelioration of adriamycin-induced cardiotoxicity by *Salsola kali* aqueous extract is mediated by lowering oxidative stress. *Redox Rep.* **2014**, *19*, 170-178.
50. Gannoun, S.; Mahfoudhi, A.; Flamini, G.; Helal, A.; Mighri, Z. Chemical composition and antimicrobial activities of Tunisian *Salsola vermiculata* L. *J. Chem. Pharm. Res.* **2016**, *8*, 1087-1092.
51. Mohammed, H.A.; Al-Omar, M.S.; Aly, M.S.; Hegazy, M.M. Essential oil constituents and biological activities of the halophytic plants, *Suaeda vermiculata* Forssk and *Salsola cyclophylla* Bakera growing in Saudi Arabia. *J. Essent. Oil Bear. Plants* **2019**, *22*, 82-93.
52. Narantuyaa, S.; Batsuren, D.; Batirov, É.K.; Malikov, V. Chemical study of plants of the Mongolian flora coumarins of *Salsola laricifolia*. *Chem. Nat. Compd.* **1986**, *22*, 228-229.
